# Factors associated with HIV pre-exposure prophylaxis use among Asian men who have sex with men in Sydney and Melbourne, Australia: a cross-sectional study

**DOI:** 10.1016/j.lanwpc.2024.101071

**Published:** 2024-04-18

**Authors:** Warittha Tieosapjaroen, Limin Mao, Horas Wong, Sujith Kumar Prankumar, Eric P.F. Chow, Christopher K. Fairley, Tiffany R. Phillips, Lei Zhang, Jason J. Ong

**Affiliations:** a Central Clinical School, Monash University, Carlton, Australia; b Melbourne Sexual Health Centre, Alfred Health, Melbourne, Australia; c Centre for Social Research in Health, University of New South Wales, Sydney, Australia; d Susan Wakil School of Nursing and Midwifery, University of Sydney, Sydney, Australia; e Kirby Institute, University of New South Wales, Sydney, Australia; f Centre for Epidemiology and Biostatistics, Melbourne School of Population and Global Health, The University of Melbourne, Melbourne, Australia; g Faculty of Infectious and Tropical Diseases, London School of Hygiene and Tropical Medicine, London, United Kingdom

**Keywords:** HIV/AIDS, Migrant, Pre-exposure prophylaxis, Public health, Health policy, International migrant, Asian-born, Men who have sex with men

## Abstract

**Background:**

Asian-born MSM are a priority population as Australia aims to end HIV transmission, but they reported additional barriers to access PrEP and other HIV prevention methods. This study investigates factors associated with PrEP use among Asian MSM in Sydney and Melbourne, Australia, to inform strategies to improve PrEP uptake in this population.

**Methods:**

This was a sub-analysis of a community-based cross-sectional survey conducted from March to June 2021. We recruited participants online in Sydney and Melbourne, Australia. Univariable and multivariable logistic regression analyses were performed to identify the factors associated with PrEP use in the last six months and lifetime. Latent class analyses were used to identify subgroups of Asian MSM sharing similar characteristics related to their risk practices for HIV.

**Findings:**

Overall, 870 Asian MSM were included: 288 Oceanian-born Asian MSM and 582 Asian-born MSM. Three latent classes were identified: 1) Asian-born MSM who recently arrived in Australia with limited English, were less likely to use PrEP and at higher risk of HIV infection (e.g., had condomless anal sex with a casual sex partner in the last six months) (4.6%); 2) Asian MSM who were at lower risk of HIV infection and less likely to use PrEP (69.3%) and; 3) Asian MSM who were at substantial risk of HIV infection and more likely to use PrEP (26.1%). Compared to Oceanian-born Asian MSM, those who were born in Southeast Asia (adjusted odds ratio (aOR) = 0.5, 95% confidence interval (CI) 0.3–0.7) and South Asia (aOR = 0.4, 95% CI 0.2–0.8) were less likely to ever use PrEP. Compared to Oceanian-born Asian MSM, those who were born in Southeast Asia (aOR = 0.4, 95% CI 0.3–0.7), Northeast Asia (aOR = 0.5, 95% CI 0.3–0.8) and South Asia (aOR = 0.4, 95% CI 0.2–0.7) were less likely to use PrEP in the last six months.

**Interpretation:**

To end HIV transmission in Australia, it will be necessary to develop strategies to improve PrEP access for the significant minority of Asian-born MSM who are at substantial risk of HIV infection.

**Funding:**

EPFC and JJO are supported by an Australian National Health and Medical Research Council (NHMRC) Emerging Leadership Investigator Grant (EPFC: GNT1172873 and JJO: GNT1193955). CKF is supported by an Australian National Health and Medical Research Council (NHMRC) Leadership Investigator Grant (GNT1172900).

**Research in context**

**Evidence before this study**

Pre-exposure prophylaxis (PrEP) programs contributed to the reduction of new HIV cases in Australia. However, HIV notifications have increased among Asian-born men who have sex with men (MSM) in Australia. This population reported additional barriers to access HIV prevention services, including PrEP. Understanding factors associated with PrEP utilisation among Asian MSM is key towards establishing effective strategies for improving PrEP accessibility among this population and reducing HIV transmission in Australia.

**Added value of this study**

This analysis demonstrates the vulnerability to HIV infection among Asian-born MSM, compared to Oceanian-born Asian MSM, evidenced by the association between their lack of PrEP experience and countries of origin (i.e., Southeast Asia, Northeast Asia and South Asia). We identified a minority (4.6%) who were less likely to use PrEP but were highly vulnerable to HIV infection. This group includes those who had recently arrived in Australia, had limited English proficiency, had more than one sexual partner and had condomless anal sex with a casual sex partner in the last six months.

**Implications of all the available evidence**

We highlight disparities in PrEP accessibility among Asian-born MSM, which can be mitigated by implementing tailored strategies, subsidised PrEP and integrated STIs and HIV services to effectively reduce HIV transmission in this population.

## Introduction

In the past decade, there was a 45% decline in HIV notifications among men who had sex with men (MSM) in Australia, from 745 cases in 2012 to 332 in 2021. Despite an overall decline in HIV notifications among MSM in Australia, HIV notifications increased 54% among Asian-born MSM from 106 in 2012 to 163 in 2019, before the start of the COVID-19 pandemic.^1^ In the previous study, compared to Australian-born MSM, Asian-born MSM were less likely to report risk behaviours (e.g., high number of sexual partners, condomless sex and injecting drug use), less likely to have ever tested for HIV and lower median CD4 count at diagnosis. It was estimated that 25% and 32% of the participants acquired HIV in Australia and overseas, respectively.^2^ In the 2021 “Gay Asian Men's community survey”, 68% of Asian MSM participants reported testing for HIV in the past six months, compared to 35% of those who reported Pre-exposure prophylaxis (PrEP) use in the past six months.^3^

PrEP is a safe and highly effective HIV biomedical prevention method for men and transgender women who have with men when users are fully adherent.^4^^,^^5^ PrEP reduced HIV transmission at the State level in Australia; a 12-month PrEP implementation trial in New South Wales (NSW) resulted in reductions in HIV notifications among MSM in 2016.^6^ The same year, the Australian Therapeutic Goods Administration (TGA) approved PrEP as part of a national plan to end HIV transmission in Australia.^7^ In in-depth qualitative studies, Asian-born MSM in Australia reported six main reasons for not using PrEP: 1) insufficient knowledge of HIV and PrEP in their country of origin; 2) stigma caused by healthcare workers; 3) unaffordable PrEP with no government subsidy; 4) struggles to navigate healthcare, Medicare and private health insurance system in Australia; 5) language barriers; and 6) negative attitudes toward PrEP users in their community.^8^^,^^9^ In this analysis of PrEP use over the first year among racial and ethnic minority individuals in the US, 41% of those were assigned to the sustained PrEP use trajectory, indicating that reducing barriers to PrEP can increase effective PrEP persistence among this vulnerable population.

PrEP was listed under the Pharmaceutical Benefits Scheme (PBS) in 2018 for Medicare-eligible individuals at risk of HIV infection.^7^ Medicare is Australia's universal health insurance program for citizens and permanent residents. Medicare-ineligible individuals, such as those on temporary student or work visas, cannot access subsidised PrEP (starting from AU$14 per 90 days' supply)^10^ and must instead, purchase PrEP at full price (starting from AU$926 per month)^11^ or import PrEP at a lower cost from online overseas pharmacies (starting from AU$18 per month).^12^ Additionally, they bear additional costs related to monitoring (e.g., tests for sexually transmitted infection (STI) and kidney function) and medical consultations. Further, PrEP policies and conventional large-scale PrEP campaigns in Australia often lack creative approaches to reach Asian MSM. We found only one PrEP education campaign specifically targeting Asian MSM who speak English as a second language. The campaign, led by the AIDS Council of NSW (ACON), an LGBTQ + community health organisation, involved Chinese influencers who identified as gay.^13^

During the COVID-19 pandemic in Australia, MSM reported reduced PrEP use due to the lower number of casual sex partners.^14^ Intriguingly, despite known barriers, a survey conducted among Asian MSM living in NSW reported an increase in PrEP use in the last six months, from 25% in 2018 to 35% in 2021 during COVID-19 pandemic. The “Gay Asian men's community survey” was part of a repeated cross-sectional survey series. Therefore, sample characteristics may vary over time, particularly during the COVID pandemic where there were restrictions on travel.^3^

Using data from Australia's largest community survey of gay Asian men, the aim of this study is to investigate factors associated with PrEP use among Asian MSM in Australia to inform strategies to improve PrEP uptake in this population.

## Methods

This paper presents a sub-analysis of the triennial “Gay Asian Men's Community Survey” conducted in Sydney and Melbourne, Australia, between March and June 2021.^3^^,^^15^ The full research method was explained elsewhere.^3^ We recruited participants online via Facebook advertisements, community organisations and health services such as ACON and the Multicultural HIV and Hepatitis Service, and were assisted by word-of-mouth among colleagues and community members. The survey was developed using insights from the annual “Gay Community Periodic Surveys” and through consultation with community members and key stakeholders, with a particular focus on exploring the intersectionality of sexuality, culture and migration. The survey was conducted in English, Traditional and Simplified Chinese and Thai. The University of NSW Human Research Ethics Committee approved the study (HC15434).

### Study populations and subpopulations

The Asian MSM community in Australia comprises Asian MSM born in Asia, Australia, and elsewhere. We defined ‘Asian MSM as self-reported gay-, bisexual-, non-binary men or queer who have sex with men and considered their ethnicity to be either Southeast, Northeast or South Asia. In this analysis, we included Asian MSM who were at least 18 years old and had no known prior HIV diagnosis. We excluded individuals if they reported: (1) living with HIV; (2) only attracted to women; (3) being bisexual women; or (4) being female at birth. Regions of birth were categorised as per the Standard Australian Classification of Countries (SACC).^16^ We defined ‘recently arrived individuals’ as individuals who arrived in Australia less than five years from the completion of the survey. Low-income earners were those whose income was lower than the poverty line (below AUD$500 per week).^17^ Regarding the sample size, with about 50% of men having ever used PrEP and a risk factor for PrEP use presenting in 30% of PrEP users, about 800 men in the study would detect a minimal odds ratio of 1.5.^3^

### Statistical analysis

We reported the proportion of Asian MSM who used PrEP in the past six months and lifetime. Univariable and multivariable logistic regression analyses were performed to identify the factors associated with PrEP use in the last six months and lifetime. Potential confounding factors (P ≤ 0.2 in univariable analyses) were included in the multivariable analyses following stepwise backward elimination (alpha = 0.2),^18^ checking the correlation between included variables (variance inflation factor>10)^19^ and expert consultation. We adjusted for age, region of birth, Medicare eligibility, English language confidence, weekly income, number of male sexual partners in the last six months, condom use when having anal sex with a casual sex partner in the last six months, and STI diagnosis in the last 12 months. Crude and adjusted odds ratios (aOR) and the corresponding 95% confidence intervals (CIs) were reported. Subgroups with similar HIV risk profiles (e.g., had more than one sexual partner, had STI diagnosis in the past six months, and had condomless anal sex with hook-ups) were identified through latent class analyses, with the number of classes determined based on convergence, interpretability, and the lowest Akaike's information criterion (AIC), Bayesian information criterion (BIC), entropy and a discussion with biostatistician and experts in public health (Table S1). Statistical analyses were performed using Stata (version 18.0, Stata Corp LP, College Station, TX, USA).

### Role of the funding source

The funding source had no involvement in this study.

## Results

Overall, 970 Asian MSM completed the survey. We excluded 100 participants who were living with HIV (n = 51), only attracted to women (n = 11), bisexual women (n = 13), and not Asian (n = 25). We included 870 Asian MSM, with 34.0% (296/870) born in Oceania and 66.0% (574/870) born in Asia. The median age was 30 (interquartile range 26–36). There were 83.5% (726/870) Asian MSM who were Medicare eligible, 8.6% (75/870) had limited English proficiency, 21.1% (184/870) recently arrived in Australia, 44.4% (386/870) had more than one male sexual partner, 25.5% (222/870) had condomless anal sex with a casual sex partner in the last six months, and 12.4% (108/870) were diagnosed with an STI in the last 12 months. The demographic characteristics are shown in Table 1.

**Table 1:** Factor associated with ever using PrEP among Asian MSM in Sydney and Melbourne, Australia (N = 870).

Variables	Ever used PrEP (Yes/All)	%	OR	95% confidence interval	P-value	S-value	aOR	95% confidence interval	P-value	S-value
**Total**	406/870	46.7								
**Age group (years)**			0.86	0.77–1.02	0.0981^a^	3.3496	0.88	0.74–1.04	0.1441^a^	2.7949
18–25	82/183	44.8	ref				ref			
26–35	223/453	49.2	1.19	0.85–1.69	0.3128	1.6767	1.17	0.77–1.77	0.4634	1.1097
36–45	79/157	50.3	1.25	0.81–1.91	0.3106	1.6869	1.44	0.82–2.54	0.2041	2.2927
46–55	13/49	26.5	0.44	0.22–0.89	0.0229	5.4485	0.48	0.20–1.15	0.1011	3.3061
>55	9/28	32.1	0.58	0.25–1.35	0.2113	2.2426	0.70	0.25–1.95	0.4927	1.0212
**Region of birth**			0.89	0.78–1.03	0.1314^a^	2.9280	0.86	0.72–1.02	0.0736^a^	3.7642
Oceania	158/296	53.4	ref				ref			
Southeast Asia	114/288	39.6	0.57	0.41–0.79	0.0009	10.1178	0.46	0.29–0.73	0.0010	9.9658
North-East Asia	101/211	47.9	0.80	0.56–1.14	0.2213	2.1759	0.65	0.40–1.04	0.0749	3.7389
South Asia and other Asian regions	33/75	44.0	0.69	0.41–1.14	0.1479	2.7573	0.37	0.19–0.72	0.0033	8.2433
**Medicare eligibility**										
Yes	352/726	48.5	ref				ref			
No	54/144	37.5	0.64	0.44–0.92	0.0163	5.9390	0.70	0.42–1.17	0.1791	2.4812
**English confidence**			1.00	0.999–1.004	0.1797^a^	2.4763	1.00	1.00–1.01	0.0818^a^	3.6118
Yes	172/360	47.8	ref				ref			
No	21/75	28.0	0.43	0.25–0.73	0.0021	8.8954	0.60	0.30–1.19	0.1422	2.8140
Unknown	213/435	49.0	1.05	0.79–1.39	0.7387	0.4369	1.20	0.84–1.71	0.3125	1.6781
**Weekly income**			1.11	1.02–1.21	0.7091^a^	0.4959	1.00	1.00–1.01	0.2972^a^	1.7505
Low (<AU$500)	82/189	43.9	ref				ref			
Middle (AU$500–1499)	176/410	42.9	0.96	0.68–1.37	0.8327	0.2641	0.89	0.58–1.35	0.5767	0.7941
High (AU$>1500)	127/230	55.2	1.58	1.07–2.33	0.0212	5.5598	1.30	0.79–2.14	0.2933	1.7696
Unknown	21/43	48.8	1.22	0.63–2.37	0.5536	0.8531	1.37	0.59–3.18	0.4602	1.1197
**Number of male sexual partners in the past 6 months**			1.96	1.63–2.45	<0.0001^a^	>13.2877	1.72	1.29–2.28	0.0002^a^	12.2877
None	35/119	29.41%	ref				ref			
One	142/365	38.90%	1.53	0.98–2.34	0.0629	3.9908	1.19	0.71–2.00	0.5069	0.9802
Multiple	229/386	59.33%	3.50	2.25–5.45	<0.0001	>13.2877	2.32	1.28–4.21	0.0058	7.4297
**Condom use when having anal sex with casual sex partners**			0.99	0.99–1.00	<0.0001^a^	>13.2877	1.00	1.00–1.01	0.1765^a^	2.5023
Always	27/96	28.1	ref				ref			
Not always	161/222	72.5	6.74	3.96–11.50	<0.0001	>13.2877	5.97	3.30–11.12	<0.0001	>13.2877
No anal sex in the past 6 months	5/28	17.9	0.56	0.19–1.61	0.2792	1.8406	0.57	0.18–1.86	0.3578	1.4828
No casual sex partners	213/524	40.7	1.75	1.09–2.82	0.0217	5.5262	3.83	2.08–7.05	<0.0001	>13.2877
**STI diagnosis in the last 12 months**			0.98	0.98–0.99	<0.0001^a^	>13.2877	0.98	0.98–0.99	<0.0001^a^	>13.2877
No	180/277	65.0	ref				ref			
Yes	87/108	80.6	2.23	1.31–3.82	0.0034	8.2002	2.00	1.11–3.61	0.0215	5.5395
Unknown	139/485	28.7	0.22	0.16–0.30	<0.0001	>13.2877	0.21	0.15–0.31	<0.0001	>13.2877

OR, odd ratio, aOR, adjusted odd ratio, STI, Sexually transmitted infection, AU$, Australian dollars, PrEP, pre-exposure prophylaxis for HIV.

a Global P-value.

### Lifetime PrEP use

Among Asian MSM reported *ever using PrEP*, 53.4% (158/296) were born in Oceania, 43.2% (248/574) were born in Asia, 48.5% (352/726) were Medicare-eligible, and 37.5% (54/144) were Medicare-ineligible (Table 1).

Table 1 and Table S2 show the multivariable model for the association between the characteristics of Asian MSM and lifetime PrEP use. Compared to Oceanian-born Asian MSM, those who were born in Southeast Asia (aOR = 0.46, 95% CI 0.29–0.73), or South Asia and other Asian regions (aOR = 0.37, 95% CI 0.19–0.72) were less likely to use PrEP. In contrast, compared to those without a sexual partner, those who have had more than one sexual partner (aOR = 2.32, 95% CI 1.28–4.21) were twice as likely to use PrEP. Similarly, compared to those who had no STI diagnosis in the last 12 months, those who had an STI diagnosis were also twice as likely to use PrEP (aOR = 2.00, 95% CI 1.11–3.61). Compared to Asian MSM who always use condoms with casual sex partners in the last six months, those who had condomless anal sex (aOR = 5.97, 95% CI 3.30–11.12) and those who had no such partner (aOR = 3.83, 95% CI 2.08–7.05) were six times and nearly quadruple as likely to use PrEP, respectively. Age, income, Medicare eligibility, and English confidence level were not statistically significantly associated with lifetime PrEP use in the multivariable analysis.

### PrEP use in the last six months

Among Asian MSM reporting using PrEP in the last six months, 44.5% (138/310) used daily PrEP and 55.5% (172/310) used on-demand PrEP (i.e., taking PrEP medications before and after a period of anticipated HIV exposure), 41.2% (122/296) were born in Oceania, 32.8% (188/574) were born in Asia, 36.6% (266/726) were Medicare-eligible, and 30.6% (44/144) were Medicare-ineligible (Table 2).

**Table 2:** Factor associated with using PrEP in the last six months among Asian MSM living in Sydney and Melbourne (N = 870).

Variables	used PrEP in the last six months (Yes/All)	%	OR	95% confidence interval	P-value	S-value	aOR	95% confidence interval	P-value	S-value
**Total**	310/870	35.6								
**Age group (Years)**			0.96	0.82–1.11	0.5657^a^	0.8219	0.97	0.81–1.17	0.7855^a^	0.3483
18–25	61/183	33.3	ref				ref			
26–35	168/453	37.1	1.18	0.82–1.69	0.3723	1.4255	1.29	0.83–1.99	0.2588	1.9501
36–45	61/157	38.9	1.27	0.81–1.98	0.2904	1.7839	1.84	1.01–3.35	0.0472	4.4051
46–55	12/49	24.5	0.65	0.32–1.33	0.2387	2.0667	0.98	0.39–2.44	0.9588	0.0607
>55	8/28	28.6	0.80	0.33–1.92	0.6174	0.6957	1.30	0.43–3.97	0.6436	0.6358
**Region of birth**			0.90	0.78–1.05	0.1809^a^	2.4667	0.86	0.72–1.02	0.0100^a^	6.6439
Oceania	122/296	41.2	ref				ref			
Southeast Asia	87/288	30.2	0.62	0.44–0.87	0.0057	7.4548	0.43	0.26–0.70	0.0007	10.4804
North-East Asia	75/211	35.6	0.79	0.55–1.13	0.1969	2.3445	0.50	0.30–0.83	0.0070	7.1584
South Asia and other Asian regions	26/75	34.7	0.76	0.45–1.28	0.3017	1.7288	0.37	0.19–0.73	0.0042	7.8954
**Medicare eligibility**										
Yes	266/726	36.6	ref				ref			
No	44/144	30.6	0.76	0.52–1.12	0.1646	2.6030	0.79	0.66–0.95	0.6661	0.5862
**English confidence**			1.00	0.998–1.004	0.4018^a^	1.3155	1.00	1.00–1.01	0.1173^a^	3.0917
Yes	131/360	36.4	ref				ref			
No	18/75	24.0	0.55	0.31–0.98	0.0417	4.5838	0.75	0.36–1.56	0.4434	1.1733
Unknown	161/435	37.0	1.03	0.77–1.37	0.8562	0.2240	1.13	0.79–1.63	0.5058	0.9834
**Weekly income**			1.00	1.00–1.01	0.1242^a^	3.0093	1.01	1.00–1.01	0.0226^a^	5.4675
Low (<AU$500)	69/1887	36.9	ref				ref			
Middle (AU$500–1499)	130/410	31.7	0.79	0.55–1.14	0.2125	2.2345	0.64	0.41–1.00	0.0507	4.3019
High (AU$>1500)	91/230	39.7	1.12	0.75–1.67	0.5776	0.7919	0.75	0.45–1.26	0.2781	1.8463
Unknown	20/43	46.5	1.49	0.76–2.90	0.2449	2.0297	1.78	0.74–4.25	0.1972	2.3423
**Number of male sexual partners in the past 6 months**			2.47	1.97–3.10	<0.0001^a^	13.2877	2.16	1.58–2.94	<0.0001^a^	13.2877
None	18/119	15.1%	ref				ref			
One	100/365	27.4%	2.12	1.22–3.68	0.0077	7.0209	1.79	0.98–3.30	0.0599	4.0613
More than one	192/386	49.7%	5.55	3.24–9.53	<0.0001	13.2877	3.89	1.99–7.63	<0.0001	13.2877
**Condom use when having anal sex with a casual sex partner**			0.99	0.989–0.995	<0.0001^a^	13.2877	1.00	1.00–1.01	0.3232^a^	1.6295
Always	19/96	19.8	ref				ref			
Not always	141/222	63.5	7.05	3.98–12.49	<0.0001	13.2877	6.00	3.20–11.24	<0.0001	13.2877
No anal sex in the past 6 months	2/28	7.1	0.31	0.07–1.43	0.1337	2.9029	0.29	0.06–1.47	0.1350	2.8890
No casual sex partners	148/524	28.2	1.60	0.93–2.72	0.0882	3.5031	3.80	1.97–7.33	<0.0001	13.2877
**STI diagnosis in the last 12 months**			0.98	0.98–0.99	<0.0001^a^	13.2877	0.98	0.98–0.99	<0.0001^a^	13.2877
No	145/277	52.4	ref				ref			
Yes	75/108	69.4	2.07	1.29–3.32	0.0026	8.5873	1.73	1.01–2.93	0.0413	4.5977
Unknown	90/485	18.6	0.21	0.15–0.29	<0.0001	13.2877	0.21	0.14–0.30	<0.0001	13.2877

OR, odd ratio, aOR, adjusted odd ratio, STI, Sexually transmitted infection, AU$, Australian dollars, ref, reference level, PrEP, pre-exposure prophylaxis for HIV.

a Global P-value.

Table 2 and Table S3 show the multivariable model for the association between Asian MSM characteristics and PrEP use in the last six months. Compared to Oceanian-born, Asian MSM born in Southeast Asia (aOR = 0.43, 95% CI 0.26–0.70), Northeast Asia (aOR = 0.50, 95% CI 0.30–0.83), and South Asia and other Asian regions (aOR = 0.37, 95% CI 0.19–0.73) were less likely to use PrEP. Meanwhile, compared to Asian MSM who always used condoms with hook-ups in the last six months, those who had condomless anal sex (aOR = 6.00, 95% CI 3.20–11.24) and those who had no such partner (aOR = 3.80, 95% CI 1.97–7.33) were six times and nearly four times as likely to PrEP, respectively. Compared to Asian MSM with no STI diagnosis, those who had an STI diagnosis in the last 12 months (aOR = 1.73, 95% CI 1.01–2.93) were more likely to use PrEP. Compared to those without a sexual partner, those who had more than one male sexual partner in the last six months (aOR = 3.89, 95% CI 1.99–7.63) were nearly quadruple as likely to use PrEP.

### Latent class analysis of PrEP use patterns

**Fig. 1:**
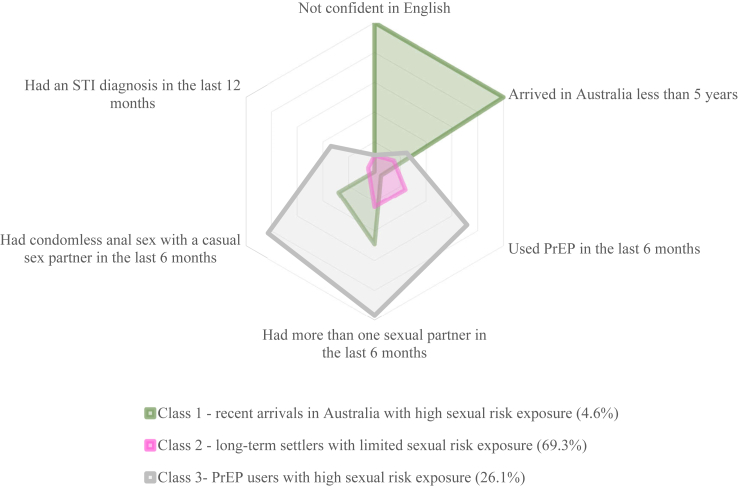
Latent class analysis for Asian men who have sex with men in Australia (N = 870).

In the latent class analysis, participants were assigned to one out of three classes based on their highest estimated probability in relation to the variables utilised. Individuals in class one, a minority group (4.6%) called *‘recent arrivals with high sexual risk exposure’*, were more likely to be recent arrivals in Australia, who were not confident in English, did not use PrEP in the last six months, had more than one male sexual partner, and had condomless anal sex with a casual sex partner in the last six months. Most participants (69.3%) were likely to be in class two, called ‘*long-term settlers with limited sexual risk exposure*’. They were more likely to arrive in Australia at least five years, have competent English, have one sexual partner, and always use condoms in anal sex with a casual sex partner, but they were less likely to use PrEP in the last six months and have had STI diagnosis in the last 12 months. Meanwhile, 26.1% of the participants were likely to be in class three, called ‘*PrEP users with high sexual risk exposure’*. They were more likely to have competent English, have arrived in Australia at least five years, have used PrEP in the last six months, have had more than one male sexual partner, have had condomless anal sex with a casual sex partner in the last six months, and have had no STI diagnosis in the last 12 months (Fig. 1 and Table S4). The sensitivity analysis showed that 29 (3%) individuals had a high probability of being in multiple classes (Table S5).

**Fig. 2:**
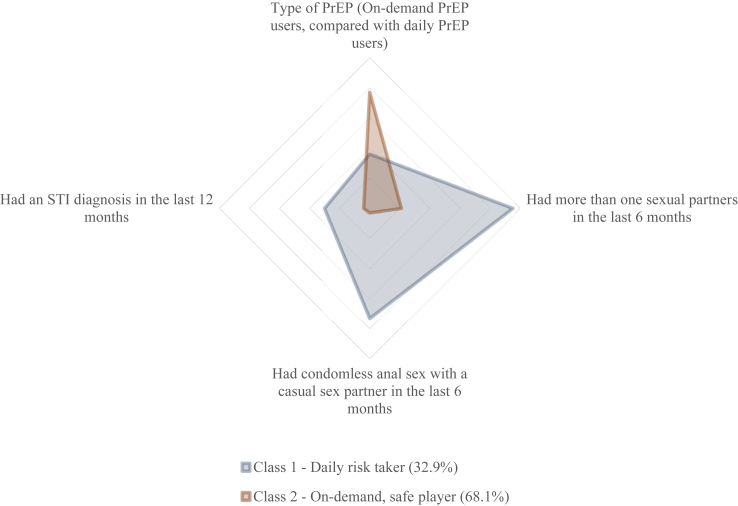
Latent class analysis of Asian men who have sex with men in Australia who are using pre-exposure prophylaxis in the last six months (N = 310).

When exploring the interaction between types of PrEP (i.e., daily and on-demand PrEP) and risk of HIV infection (i.e., STI diagnosis, more than one sexual partner, condomless anal sex with a casual sex partner) among Asian MSM who used PrEP in the last six months, two classes were identified. Most participants (68.1%) were likely to be in class two, called ‘*On-demand, safe player’*. They were more likely to use on-demand PrEP, have one sexual partner, use condoms in anal sex with a casual sex partner in the last six months and have had no STI diagnosis in the last 12 months. Meanwhile, 32.9% of the participants were likely to be in class one, called ‘*Daily risk takers’*. They were more likely to use daily PrEP, have more than one sexual partner, have condomless anal sex with a casual sex partner, and have no STI diagnosis in the last six months (Fig. 2 and Table S6). The sensitivity analysis showed that 25 (3%) individuals had a high probability of being in multiple classes (Table S7).

## Discussion

This analysis demonstrates the vulnerability to HIV infection among Asian-born MSM, compared to Oceanian-born, evidenced by the association between their lack of PrEP experience and countries of origin (i.e., Southeast Asia, Northeast Asia and South Asia). Through the latent class analysis, we identified a minority (4.6%) who were less likely to use PrEP but were highly vulnerable to HIV infection. This group includes those who had recently arrived in Australia, had limited English proficiency, had more than one sexual partner and had condomless anal sex with a casual sex partner in the last six months. Our analysis's findings emphasise the importance of programs tailored to improve PrEP uptake among Asian-born MSM, a population at substantial risk of HIV infection in Australia.

To achieve Australia's goal of eliminating HIV transmission by 2030, our analyses indicate that bridging PrEP accessibility gaps, irrespective of individuals' countries of origin, and Medicare eligibility, should be a priority. A previous study reported that overseas-born individuals encountered additional barriers in accessing PrEP, including limited knowledge of HIV and PrEP in their countries of origin, navigation challenges within host healthcare systems and no affordable or subsidised PrEP, compared to individuals born in high-income countries.^20^ Similarly, our findings revealed lower PrEP utilisation among Medicare-ineligible individuals. However, Medicare eligibility was not statistically associated with PrEP use in the multivariable analyses, likely influenced by the high proportion of Medicare-eligible participants (83.5%) during COVID-19 when the international border was closed. To ensure equitable PrEP access, we need effective policies or strategies to promote affordability and equitable access for all.

Despite most Asian MSM exhibiting a lower risk of HIV infection, a group of migrant Asian MSM (4.6%) were at substantial risk of HIV infection and did not utilise PrEP. This finding amplifies a concern about the potential of HIV transmission within this population and broader communities. Tailored and creative approaches are needed to reach this population and increase PrEP uptake. Innovative approaches, such as crowdsourcing and designathons, are promising tools to engage end users and experts in generating community-based solutions to tackle unique challenges encountered by minority populations.^21^ Crowdsourcing involves engaging the crowd to solve a problem and then sharing the solution publicly.^22^ Designathons is a three-stage participatory activity informed by design thinking that includes preparation with end-users, a period of intensive collaboration, and follow–up activities for implementation and research.^23^ These methods increase public awareness of specific health challenges and foster a sense of community empowerment and collective responsibility, leading to more sustainable and impactful outcomes in health-related initiatives.^21^ These approaches have successfully generated solutions to address HIV-related challenges^24^, ^25^, ^26^ and can be cost-effective.^27^ Additionally, future research should consider differentiating factors associated with PrEP use among local and non-local Asian MSM. Employing a theoretical framework would be instrumental in providing an understanding of the influences contributing to the lower rate of PrEP utilization within non-local population.

Our findings revealed a noteworthy concern: the relationship between the history of STIs and PrEP utilisation among Asian MSM. Despite a report of increased STI incidence among MSM upon scaling up PrEP in Australia, STI incidence plateaued and reduced in the subsequent years, largely attributed to frequent testing practices among PrEP users.^28^^,^^29^ Recognising the significance of these interrelated patterns, it is vital to establish integrated services of PrEP provision and STIs, potentially enhancing the quality of care and contributing to a reduction in HIV and STI transmissions.^30^ Additionally, promoting holistic and positive concepts of sexual health within integrated services are needed. Promoting PrEP as a health promotion strategy may encourage people to use HIV/STI prevention methods and services more than framing PrEP as a risk reduction strategy.

Our analysis strength is using data from one of Australia's largest surveys targeting Asian MSM. There are several benefits of comparing Oceanian-born Asian MSM with Asian-born MSM. Firstly, this comparison allows for a more nuanced understanding of cultural factors (e.g., language proficiency and access to culturally relevant resources). Secondly, by considering regions of birth, the study acknowledges the intersectionality of identities and experiences among Asian MSM. This approach allows for a deeper exploration of how factors such as ethnicity, migration history, ability to access and navigate healthcare systems and cultural background intersect to influence PrEP use and vulnerability to HIV. Our study underscores the importance of addressing structural barriers and promoting culturally tailored interventions to improve PrEP access and uptake among Asian MSM.

However, we acknowledge some limitations. First, the exclusive online conduct of our survey during COVID-19 restrictions and the closure of international borders led to a reduced participation rate among overseas-born individuals, declining from 82% in the 2018 survey to 62% in 2021. Additionally, MSM reported changes in sexual practices and PrEP utilisation during COVID-19.^14^ Second, our reliance on non-probabilistic sampling methods for recruitment, targeting specific populations, potentially limits the generalisability of our findings to the broader Asian MSM population. We mitigated these limitations by exploring the association between PrEP use and demographic characteristics, allowing readers to assess the sample's representativeness. However, we acknowledge that our sample underrepresents populations who have limited digital literacy or internet access, which were likely to be older people who were less sexually active. Third, unrealistically large odds ratio confidence interval arose from sparse-data bias due to the sample sizes. Future studies need larger sample sizes to mitigate the impact of sparse-data bias.^31^ Fourth, despite our efforts to control for known confounders, the possibility of unmeasured confounding exists. Future research could benefit from a more comprehensive examination of potential confounders to enhance the robustness of findings. Fifth, certain variables were predefined as categorical not continuous. While we acknowledge this limitation, we addressed it by merging sub-categories with low denominators (N < 20) when necessary. Additionally, the effectiveness of the proposed tailored PrEP approaches for Asian-born MSM requires further validation.

In conclusion, a lower PrEP utilisation was observed among a minority highly vulnerable to HIV infection and underserved Asian MSM, including those born in specific parts of Asia, who had recently arrived in Australia and had limited English proficiency. Our analysis highlights disparities in PrEP accessibility, which can be mitigated by implementing tailored approaches, subsidised PrEP and integrated STIs and HIV services to effectively reduce HIV transmission in this population.

## Contributors

JJO and LM conceived the idea. WT analysed the data, wrote the first draft, revised it, and finalised the manuscript. All authors contributed to the manuscript and approved the final version for submission.

## Data sharing statement

The data that support the findings of this study are available on request from the authors, JO and LM. The data are not publicly available due to their containing information that could compromise the privacy of research participants.

## Declaration of interests

No authors reported conflict of interest.

## Appendix A Supplementary data

Supplementary Tables

STROBE_checklist_cross-sectional
