# Supplementary material for: Factors associated with HIV pre-exposure prophylaxis use among Asian men who have sex with men in Sydney and Melbourne, Australia: a cross-sectional study
Source: Lancet Reg Health West Pac. 2024 Apr 18;46:101071. doi: 10.1016/j.lanwpc.2024.101071 (PMC11047000; doi:10.1016/j.lanwpc.2024.101071)
Supplement: Supplementary Tables [file mmc1.docx]

**Table S1. Evaluating class solutions**

| **Variables included** | **Number of classes** | **Model fit criteria** | | **Diagnostic criteria** | **Interpretability** |
| --- | --- | --- | --- | --- | --- |
|  |  | **AIC** | **BIC** | **Entropy** |  |
| PrEP dose  Number of sexual partner  Condom use with a casual sex partner  STI diagnosis in the past six months | 2 | 2908.583 | 2951.500 | 0.72639969 | Meaningful |
| PrEP dose  Number of sexual partner  Condom use with a casual sex partner  STI diagnosis in the past six months | 3 | Convergence not achieved |  |  |  |
| PrEP use in lifetime  Number of sexual partner  Condom use with a casual sex partner  STI diagnosis in the past six months | 2 | 3619.366 | 3662.282 | 0.8105302 | Not meaningful |
| PrEP use in lifetime  Number of sexual partner  Condom use with a casual sex partner  STI diagnosis in the past six months | 3 | 3621.385 | 3688.144 | 0.43781174 | Not meaningful |
| PrEP use in lifetime  Number of sexual partner  Condom use with a casual sex partner  STI diagnosis in the past six months | 4 | Convergence not achieved |  |  |  |
| English fluency  Length of stay in Australia  PrEP use in the past six months  Number of sexual partner  Condom use with a casual sex partner  STI diagnosis in the past six months | 3 | 4799.256 | 4894.626 | 0.86132 | Meaningful |
| English fluency  Length of stay in Australia  PrEP use in the past six months  Number of sexual partner  Condom use with a casual sex partner  STI diagnosis in the past six months | 4 | Convergence not achieved |  |  |  |

**Table S2. Univariate regression analysis of factors associated with ever PrEP use among Asian MSM**

| **Variables** | **Ever used PrEP**  **(Yes/N)** | **%** | **OR** | **95CI** | **P-value** |
| --- | --- | --- | --- | --- | --- |
| **Demographic characteristics** |  |  |  |  |  |
| **Age group (years)** |  |  | 0.86 | 0.77-1.02 | 0.098 ^G^ |
| 18-25 | 82/183 | 44·8 | ref |  |  |
| 26-35 | 223/453 | 49·2 | 1·19 | 0·85-1·69 | 0·313 |
| 36-45 | 79/157 | 50·3 | 1·25 | 0·81-1·91 | 0·311 |
| 46-55 | 13/49 | 26·5 | 0·44 | 0·22-0·89 | 0·023 |
| >55 | 9/28 | 32·1 | 0·58 | 0·25-1·35 | 0·211 |
|  |  |  |  |  |  |
| **Birthplace** |  |  |  |  |  |
| Australia | 152/288 | 52.8 | ref |  |  |
| Asian | 254/582 | 43.6 | 0.69 | 0.52-0.92 | 0.011 |
|  |  |  |  |  |  |
| **Region of birth** |  |  | 0.89 | 0.78-1.03 | 0.131 ^G^ |
| Oceania | 158/296 | 53·4 | ref |  |  |
| Southeast Asia | 114/288 | 39·6 | 0·57 | 0·41-0·79 | 0·001 |
| North-East Asia | 101/211 | 47·9 | 0·80 | 0·56-1·14 | 0·221 |
| South Asia and other Asian regions | 33/75 | 44·0 | 0·69 | 0·41-1·14 | 0·148 |
|  |  |  |  |  |  |
| **Ethnicity** |  |  | 1.00 | 0.996-1.005 | 0.827 ^G^ |
| Chinese | 147/349 | 42.5 | ref |  |  |
| Thai | 55/95 | 57.9 | 1.86 | 1.18-2.95 | 0.008 |
| Indian | 40/83 | 48.2 | 1.26 | 0.78-2.04 | 0.347 |
| Malaysian | 20/37 | 54.1 | 1.59 | 0.81-3.15 | 0.180 |
| Vietnamese | 26/61 | 42.6 | 1.01 | 0.58-1.74 | 1.984 |
| Filipino | 51/103 | 49.5 | 1.33 | 0.85-2.06 | 0.208 |
| Other | 39/76 | 51.3 | 1.16 | 0.68-1.98 | 0.584 |
|  |  |  |  |  |  |
| **Medicare eligibility** |  |  |  |  |  |
| Yes | 352/726 | 48.5 | ref |  |  |
| No | 54/144 | 37.5 | 0.64 | 0.44-0.92 | 0.016 |
|  |  |  |  |  |  |
| **Citizen/Permanent resident** |  |  | 0.99 | 0.97-1.00 | 0.133 ^G^ |
| Yes | 329/663 | 49.6 | ref |  |  |
| No | 75/198 | 37.9 | 0.62 | 0.45-0.86 | 0.004 |
| Unknown | 2/9 |  | 0.29 | 0.06-1.41 | 0.124 |
|  |  |  |  |  |  |
| **Length of living in Australia** |  |  |  |  |  |
| At least 5 years | 333/686 | 48.5 | ref |  |  |
| Less than 5 years | 73/184 | 39.7 | 0.70 | 0.50-0.97 | 0.033 |
|  |  |  |  |  |  |
| **English confidence** |  |  | 1.00 | 0.999-1.004 | 0.180 ^G^ |
| Yes | 172/360 | 47.8 | ref |  |  |
| No | 21/75 | 28.0 | 0.43 | 0.25-0.73 | 0.002 |
| Unknown | 213/435 | 49.0 | 1.05 | 0.79-1.39 | 0.739 |
|  |  |  |  |  |  |
| **Education level** |  |  | 0.99 | 0.97-1.02 | 0.694^G^ |
| At least bachelor | 309/630 | 49.1 | ref |  |  |
| Less than bachelor | 97/240 | 40.4 | 0.70 | 0.52-0.95 | 0.023 |
|  |  |  |  |  |  |
| **Employment** |  |  | 0.98 | 0.68-1.41 | 0.921 ^G^ |
| Employed | 336/715 | 47.0 | ref |  |  |
| Student | 34/74 | 46.0 | 0.96 | 0.59-1.55 | 0.864 |
| Unemployed | 36/81 | 44.4 | 0.90 | 0.57-1.43 | 0.663 |
|  |  |  |  |  |  |
| **Weekly income** |  |  | 1.11 | 1.02-1.21 | 0.012 ^G^ |
| Low (<AU$500) | 82/189 | 43.9 | ref |  |  |
| Middle (AU$500-1,499) | 176/410 | 42.9 | 0.96 | 0.68-1.37 | 0.833 |
| High (AU$>1,500) | 127/230 | 55.2 | 1.58 | 1.07-2.33 | 0.021 |
| Unknown | 21/43 | 48.8 | 1.22 | 0.63-2.37 | 0.554 |
|  |  |  |  |  |  |
| **Religious** |  |  |  |  |  |
| Yes | 191/405 | 47.2 | ref |  |  |
| No | 215/465 | 46.2 | 0.96 | 0.74-1.26 | 0.785 |
|  |  |  |  |  |  |
| **Sexuality-related stigma** |  |  | 1.40 | 1.07-1.84 | 0.016 ^G^ |
| No or rarely | 227/525 | 43.2 | ref |  |  |
| Sometimes | 107/204 | 52.5 | 1.45 | 1.05-2.00 | 0.025 |
| Usually or always | 70/139 | 50.4 | 1.33 | 0.92-1.94 | 0.134 |
| Unknown | 2/2 |  | omitted |  |  |
|  |  |  |  |  |  |
| **Sexuality-related stigma by healthcare workers** |  |  | 1.65 | 1.21-2.28 | 0.002 ^G^ |
| No or rarely | 291/667 | 43.6 | ref |  |  |
| Sometimes | 68/123 | 55.3 | 1.60 | 1.08-2.35 | 0.018 |
| Usually or always | 45/78 | 57.7 | 1.76 | 1.10-2.83 | 0.019 |
| Unknown | 2/2 |  | omitted |  |  |
|  |  |  |  |  |  |
| **Racism** |  |  | 1.00 | 0.99-1.01 | 0.381 ^G^ |
| No or rarely | 172/415 | 41.5 | ref |  |  |
| sometimes or always | 255/439 | 51.3 | 1.49 | 1.13-1.95 | 0.004 |
| Unknown | 9/16 |  | 1.82 | 0.66-4.97 | 0.245 |
|  |  |  |  |  |  |
| **Racism from healthcare workers** |  |  | 1.00 | 0.99-1.01 | 0.418 ^G^ |
| No or rarely | 296/660 | 44.9 | ref |  |  |
| Sometimes or always | 100/192 | 52.1 | 1.34 | 0.97-1.84 | 0.077 |
| Unknown | 10/18 |  | 1.54 | 0.61-3.94 | 0.371 |
|  |  |  |  |  |  |
| **Number of male sexual partners in the past 6 months** |  |  | 1.96 | 1.63-2.45 | <0.001 ^G^ |
| None | 35/119 | 29.4 | ref |  |  |
| One | 142/365 | 38.9 | 1·53 | 0·98-2·34 | 0·063 |
| Multiple | 229/386 | 59.3 | 3·50 | 2·25-5·45 | <0·001 |
|  |  |  |  |  |  |
| **Being in a committed relationship** |  |  | 1.09 | 0.87-1.37 | 0.447 ^G^ |
| Single | 207/430 | 48.1 | ref |  |  |
| Yes | 167/399 | 41.9 | 0.78 | 0.59-1.02 | 0.069 |
| **No** | 32/41 | 78.1 | 3.83 | 1.79-8.22 | 0.001 |
|  |  |  |  |  |  |
| **Sex with PLWH in the last 6 months** |  |  | 0.85 | 0.62-1.16 | 0.294 ^G^ |
| Yes | 35/49 | 71.4 | ref |  |  |
| No | 275/587 | 46.9 | 0.35 | 0.19-0.67 | 0.001 |
| Not sure | 61/115 | 53.0 | 0.45 | 0.22-0.93 | 0.031 |
|  |  |  |  |  |  |
| **Condom use when having anal sex with a casual sex partner** |  |  | 0.99 | 0.99-1.00 | <0.001 ^G^ |
| Always | 27/96 | 28.1 | ref |  |  |
| Not always | 161/222 | 72.5 | 6.74 | 3.96-11.50 | <0.001 |
| No anal sex in the past 6 months | 5/28 | 17.9 | 0.56 | 0.19-1.61 | 0.270 |
| No casual sex partners | 213/524 | 40.7 | 1.75 | 1.09-2.82 | 0.022 |
|  |  |  |  |  |  |
| **STI diagnosis in the last 12 months** |  |  | 0.98 | 0.98-0.99 | <0.001 ^G^ |
| Yes | 87/108 | 80.6 | ref |  |  |
| No | 180/277 | 65.0 | 0.45 | 0.26-0.77 | 0.003 |
| Unknown | 139/485 | 28.7 | 0.10 | 0.06-0.16 | <0.001 |
|  |  |  |  |  |  |
| **Domestic violence** |  |  | 1.00 | 0.99-1.01 | 0.507 ^G^ |
| No | 347/772 | 45.0 | ref |  |  |
| Yes | 53/82 | 64.6 | 2.24 | 1.39-3.60 | 0.001 |
| Unknown | 6/16 |  | 0.73 | 0.26-2.04 | 0.555 |
|  |  |  |  |  |  |
| **Group sex in the past 6 months** |  |  | 0.99 | 0.987-0.993 | <0.001 ^G^ |
| No | 102/206 | 49.5 | ref |  |  |
| Yes | 127/180 | 70.6 | 2.44 | 1.60-3.72 | <0.001 |
| Unknown | 177/484 | 36.6 | 0.59 | 0.42-0.82 | 0.002 |
|  |  |  |  |  |  |
| **Chem sex in the past 6 months** |  |  | 0.99 | 0.987-0.996 | <0.001 ^G^ |
| No | 268/597 | 44.9 | ref |  |  |
| Yes | 101/151 | 66.9 | 0.99 | 0.99-1.00 | <0.001 |
| Unknown | 37/122 | 30.3 | 0.97 | 0.84-1.12 | 0.717 |
|  |  |  |  |  |  |
| **Had sex in exchange for gift or money in the past 6 months** |  |  | 0.99 | 0.987-0.996 | <0.001 ^G^ |
| No | 310/659 | 47.0 | ref |  |  |
| Yes | 61/92 | 66.3 | 0.99 | 0.99-1.00 | <0.001 |
| Unknown | 35/119 | 29.4 | 0.98 | 0.85-1.13 | 0.745 |
|  |  |  |  |  |  |
| **Sex in oversea countries** |  |  | 1.25 | 0.95-1.63 | 0.107 ^G^ |
| No | 95/251 | 37.9 | ref |  |  |
| Yes | 311/619 | 50.2 | 1.66 | 1.23-2.24 | 0.001 |

^G^ Global P-value

95%CI= 95 confidence interval, AU$=Australian dollars, PLWH=people living with HIV, PrEP=pre-exposure prophylaxis, ref= reference level, STI=sexually transmitted infection

**Table S3. Univariate regression analysis of factors associated with PrEP use in the last six months among Asian MSM**

| **Variables** | **Used PrEP in the last 6 months**  **(Yes/N)** | **%** | **OR** | **95CI** | **P-value** |
| --- | --- | --- | --- | --- | --- |
| **Total** | 310/870 | 35.6 |  |  |  |
| **Age group (Years)** |  |  | 0.96 | 0.82-1.11 | 0.566 ^G^ |
| 18-25 | 61/183 | 33·3 | ref |  |  |
| 26-35 | 168/453 | 37·1 | 1·18 | 0·82-1·69 | 0·372 |
| 36-45 | 61/157 | 38·9 | 1·27 | 0·81-1·98 | 0·290 |
| 46-55 | 12/49 | 24·5 | 0·65 | 0·32-1·33 | 0·239 |
| >55 | 8/28 | 28·6 | 0·80 | 0·33-1·92 | 0·617 |
|  |  |  |  |  |  |
| **Birthplace** |  |  |  |  |  |
| Australia | 117/288 | 40.6 | ref |  |  |
| Asian | 193/582 | 33.2 | 0.73 | 0.54-0.97 | 0.031 |
|  |  |  |  |  |  |
| **Region of birth** |  |  | 0.90 | 0.78-1.05 | 0.181 ^G^ |
| Oceania | 122/296 | 41·2 | ref |  |  |
| Southeast Asia | 87/288 | 30·2 | 0·62 | 0·44-0·87 | 0·006 |
| North-East Asia | 75/211 | 35·6 | 0·79 | 0·55-1·13 | 0·197 |
| South Asia and other Asian regions | 26/75 | 34·7 | 0·76 | 0·45-1·28 | 0·302 |
|  |  |  |  |  |  |
| **Ethnicity** |  |  | 1.00 | 1.00-1.01 | 0.799 ^G^ |
| Chinese | 108/346 | 31.2 | ref |  |  |
| Thai | 44/95 | 46.3 | 1.90 | 1.20-3.02 | 0.007 |
| Indian | 32/83 | 38.6 | 1.38 | 0.84-2.27 | 0.201 |
| Malaysian | 15/37 | 40.5 | 1.50 | 0.75-3.01 | 0.251 |
| Vietnamese | 19/61 | 31.2 | 1.00 | 0.55-1.79 | 0.992 |
| Filipino | 40/103 | 38.8 | 1.40 | 0.88-2.21 | 0.150 |
| Mixed | 23/69 | 33.3 | 1.10 | 0.64-1.91 | 0.730 |
| Other | 29/76 | 38.2 | 1.36 | 0.81-2.28 | 0.243 |
|  |  |  |  |  |  |
| **Medicare eligibility** |  |  |  |  |  |
| Yes | 266/726 | 36.6 | ref |  |  |
| No | 44/144 | 30.6 | 0.76 | 0.52-1.12 | 0.165 |
|  |  |  |  |  |  |
| **Citizen/Permanent resident** |  |  | 0.99 | 0.98-1.01 | 0.365 ^G^ |
| Yes | 249/663 | 37.6 | ref |  |  |
| No | 59/198 | 29.8 | 0.71 | 0.50-0.99 | 0.046 |
| Unknown | 2/9 |  | 0.45 | 0.10-2.3 | 0.356 |
|  |  |  |  |  |  |
| **Length of living in Australia** |  |  |  |  |  |
| At least 5 years | 250/686 | 36.4 | ref |  |  |
| Less than 5 years | 60/184 | 32.6 | 0.84 | 0.60-1.19 | 0.335 |
|  |  |  |  |  |  |
| **English confidence** |  |  | 1.00 | 0.998-1.004 | 0.402 ^G^ |
| Yes | 131/360 | 36.4 | ref |  |  |
| No | 18/75 | 24.0 | 0.55 | 0.31-0.98 | 0.042 |
| Unknown | 161/435 | 37.0 | 1.03 | 0.77-1.37 | 0.856 |
|  |  |  |  |  |  |
| **Education level** |  |  |  |  |  |
| At least bachelor | 228/630 | 36.2 | ref |  |  |
| Less than bachelor | 82/240 | 34.2 | 0.92 | 0.67-1.25 | 0.578 |
|  |  |  |  |  |  |
| **Employment** |  |  | 0.86 | 0.58-1.26 | 0.435 ^G^ |
| Employed | 252/715 | 35.2 | ref |  |  |
| Student | 28/74 | 37.8 | 1.12 | 0.68-1.83 | 0.657 |
| Unemployed | 30/81 | 37.0 | 1.08 | 0.67-1.74 | 0.749 |
|  |  |  |  |  |  |
| **Weekly income** |  |  | 1.00 | 1.001.01 | 0.124 ^G^ |
| Low (<AU$500) | 69/1887 | 36.9 | ref |  |  |
| Middle (AU$500-1,499) | 130/410 | 31.7 | 0.79 | 0.55-1.14 | 0.212 |
| High (AU$>1,500) | 91/230 | 39.7 | 1.12 | 0.75-1.67 | 0.578 |
| Unknown | 20/43 | 46.5 | 1.49 | 0.76-2.90 | 0.245 |
|  |  |  |  |  |  |
| **Religious** |  |  |  |  |  |
| Yes | 152/405 | 37.5 | ref |  |  |
| No | 158/465 | 34.0 | 0.86 | 0.65-1.13 | 0.275 |
|  |  |  |  |  |  |
| **Sexuality-related stigma** |  |  | 1.24 | 0.93-1.65 | 0.136 ^G^ |
| No or rarely | 176/525 | 33.5 | ref |  |  |
| Sometimes | 82/204 | 40.2 | 1.33 | 0.95-1.86 | 0.091 |
| Usually or always | 50/139 | 36.0 | 1.11 | 0.75-1.65 | 0.588 |
| Unknown | 2/2 |  | omitted |  |  |
|  |  |  |  |  |  |
| **Sexuality-related stigma by healthcare workers** |  |  | 1.31 | 0.95-1.81 | 0.104 ^G^ |
| No or rarely | 227/667 | 34.0 | ref |  |  |
| Sometimes | 46/123 | 37.4 | 1.16 | 0.78-1.73 | 0.471 |
| Usually or always | 35/78 | 44.9 | 1.58 | 0.98-2.53 | 0.059 |
| Unknown | 2/2 |  | omitted |  |  |
|  |  |  |  |  |  |
| **Racism** |  |  | 1.00 | 0.99-1.01 | 0.801 ^G^ |
| No or rarely | 1308415 | 31.3 | ref |  |  |
| Sometimes | 105/249 | 42.1 | 1.60 | 1.15-2.21 | 0.005 |
| Usually or always | 69/190 | 36.3 | 1.25 | 0.87-1.79 | 0.226 |
| Unknown | 6/16 |  | 1.32 | 0.47-3.70 | 0.603 |
|  |  |  |  |  |  |
| **Racism from healthcare workers** |  |  | 1.00 | 0.99-1.01 | 0.715 ^G^ |
| No or rarely | 220/660 | 33.3 | ref |  |  |
| Sometimes | 46/112 | 41.1 | 1.39 | 0.93-2.10 | 0.112 |
| Usually or always | 37/80 | 46.3 | 1.72 | 1.08-2.75 | 0.023 |
| Unknown | 7/18 |  | 1.27 | 0.49-3.33 | 0.623 |
|  |  |  |  |  |  |
| **Number of male sexual partners in the past 6 months** |  |  | 2.47 | 1.97-3.10 | <0.001 ^G^ |
| None | 18/119 | 15.1 | ref |  |  |
| One | 100/365 | 27.4 | 2.12 | 1.22-3.68 | 0.008 |
| More than one | 192/386 | 49.7 | 5.55 | 3.24-9.53 | <0.001 |
|  |  |  |  |  |  |
| **Being in a committed relationship** |  |  | 0.81 | 0.63-1.03 | 0.082 ^G^ |
| Single | 174/430 | 40.5 | ref |  |  |
| Yes | 115/399 | 28.8 | 0.60 | 0.44-0.80 | <0.001 |
| No | 21/41 | 51.2 | 1.54 | 0.81-2.94 | 0.184 |
|  |  |  |  |  |  |
| **Sex with PLWH in the last 6 months** |  |  | 0.86 | 0.62-1.19 | 0.356 ^G^ |
| Yes | 32/49 | 65.3 | ref |  |  |
| No | 208/587 | 35.4 | 0.29 | 0.16-0.54 | <0.001 |
| Not sure | 52/115 | 45.2 | 0.44 | 0.22-0.88 | 0.020 |
|  |  |  |  |  |  |
| **Condom use when having anal sex with a casual sex partner in the last 6 months** |  |  | 0.99 | 0.989-0.995 | <0.001 ^G^ |
| Always | 19/96 | 19.8 | ref |  |  |
| Not always | 141/222 | 63.5 | 7.05 | 3.98-12.49 | <0.001 |
| No anal sex in the past 6 months | 2/28 | 7.1 | 0.31 | 0.07-1.43 | 0.134 |
| No casual sex partners | 148/524 | 28.2 | 1.60 | 0.93-2.72 | 0.088 |
|  |  |  |  |  |  |
| **STI diagnosis in the last 12 months** |  |  | 0.98 | 0.979-0.985 | <0.001 ^G^ |
| Yes | 75/108 | 69.4 | ref |  |  |
| No | 145/277 | 52.4 | 0.48 | 0.30-0.78 | 0.003 |
| Unknown | 90/485 | 18.6 | 0.10 | 0.06-0.16 | <0.001 |
|  |  |  |  |  |  |
| **Domestic violence** |  |  | 1.00 | 0.98-1.01 | 0.397 ^G^ |
| No | 269/772 | 34.8 | ref |  |  |
| Yes | 37/82 | 45.1 | 1.54 | 0.97-2.43 | 0.067 |
| Unknown | 4/16 |  | 0.62 | 0.20-1.95 | 0.417 |
|  |  |  |  |  |  |
| **Group sex in the past 6 months** |  |  | 0.99 | 0.985-0.992 | <0.001 ^G^ |
| No | 84/206 | 40.8 | ref |  |  |
| Yes | 108/180 | 60.0 | 2.18 | 1.45-3.27 | <0.001 |
| Unknown | 118/484 | 24.4 | 0.47 | 0.33-0.66 | <0.002 |
|  |  |  |  |  |  |
| **Chem sex in the past 6 months** |  |  | 0.99 | 0.983-0.993 | <0.001 ^G^ |
| No | 210/579 | 35.2 | ref |  |  |
| Yes | 80/151 | 53.0 | 0.99 | 0.98-0.99 | <0.001 |
| Unknown | 20/122 | 16.3 | 0.63 | 0.55-0.73 | <0.002 |
|  |  |  |  |  |  |
| **Had sex in exchange for gift or money in the past 6 months** |  |  | 0.99 | 0.98-0.99 | <0.001 ^G^ |
| No | 246/659 | 37.3 | ref |  |  |
| Yes | 46/92 | 50.0 | 0.99 | 0.98-0.99 | <0.001 |
| Unknown | 18/119 | 15.1 | 0.64 | 0.55-0.74 | <0.002 |
|  |  |  |  |  |  |
| **Sex in oversea countries** |  |  |  |  |  |
| No | 148/439 | 33.7 | ref |  |  |
| Yes | 162/431 | 37.6 | 1.18 | 0.90-1.56 | 0.233 |

95CI= 95 confidence interval, AU$=Australian dollars, PLWH=people living with HIV, PrEP=pre-exposure prophylaxis, ref= reference level, STI=sexually transmitted infection

^G^ Global P-value

**Table S4. Probability of PrEP use in the last six months and risk of HIV infection among Asian MSM in Australia**

|  | **Class 1**  **Recent arrivals in Australia with high sexual risk exposure (4.6)** | **Class 2**  **Long-term settlers with limited sexual risk exposure (69.3)** | **Class 3  Sexually experienced and STI-free (26.1)** |
| --- | --- | --- | --- |
| **Probability of** |  |  |  |
| Not confident in English | 1.00 | 0.11 | 0.11 |
| Arrived in Australia less than 5 years | 1.00 | 0.15 | 0.25 |
| Used PrEP in the last six months | 0.05 | 0.24 | 0.72 |
| Had more than one sexual partner in the last six months | 0.49 | 0.24 | 0.97 |
| Had condomless anal sex with a casual sex partner in the last six months | 0.28 | 0.04 | 0.83 |
| Had an STI diagnosis in the last twelve months | 0.00 | 0.05 | 0.34 |

Akaike’s information criterion = 4,799.256

Bayesian information criterion = 4,894.626

*STI=sexually transmitted infection, PrEP= Pre-exposure prophylaxis for HIV

**Table S5. Probability of each individual in each class for latent class for PrEP use in the past six months, length of living in Australia, English proficiency and risk of HIV infection**

| **ID** | **newly** | **gpr1** | **gpr2** | **gpr3** | **ID** | **newly** | **gpr1** | **gpr2** | **gpr3** |
| --- | --- | --- | --- | --- | --- | --- | --- | --- | --- |
| 87 | No | 0.9994 | 0.0006 | 0 | 394 | No | 0.9994 | 0.0006 | 0 |
| 797 | No | 0.9994 | 0.0006 | 0 | 49 | No | 0.9994 | 0.0006 | 0 |
| 611 | No | 0.9994 | 0.0006 | 0 | 293 | No | 0.9994 | 0.0006 | 0 |
| 61 | No | 0.9994 | 0.0006 | 0 | 37 | No | 0.9994 | 0.0006 | 0 |
| 724 | No | 0.9994 | 0.0006 | 0 | 269 | No | 0.9994 | 0.0006 | 0 |
| 9 | No | 0.9994 | 0.0006 | 0 | 733 | No | 0.9994 | 0.0006 | 0 |
| 286 | No | 0.9994 | 0.0006 | 0 | 259 | No | 0.9994 | 0.0006 | 0 |
| 105 | No | 0.9994 | 0.0006 | 0 | 151 | No | 0.9994 | 0.0006 | 0 |
| 75 | No | 0.9994 | 0.0006 | 0 | 154 | No | 0.9994 | 0.0006 | 0 |
| 242 | No | 0.9994 | 0.0006 | 0 | 563 | No | 0.9994 | 0.0006 | 0 |
| 842 | No | 0.9994 | 0.0006 | 0 | 664 | No | 0.9994 | 0.0006 | 0 |
| 937 | No | 0.9994 | 0.0006 | 0 | 607 | No | 0.9994 | 0.0006 | 0 |
| 814 | No | 0.9994 | 0.0006 | 0 | 246 | No | 0.9994 | 0.0006 | 0 |
| 893 | No | 0.9994 | 0.0006 | 0 | 276 | No | 0.9994 | 0.0006 | 0 |
| 180 | No | 0.9994 | 0.0006 | 0 | 502 | No | 0.9994 | 0.0006 | 0 |
| 45 | No | 0.9994 | 0.0006 | 0 | 878 | No | 0.9994 | 0.0006 | 0 |
| 256 | No | 0.9994 | 0.0006 | 0 | 267 | No | 0.9994 | 0.0006 | 0 |
| 137 | No | 0.9994 | 0.0006 | 0 | 911 | No | 0.9994 | 0.0006 | 0 |
| 763 | No | 0.9994 | 0.0006 | 0 | 736 | No | 0.9994 | 0.0006 | 0 |
| 719 | No | 0.9994 | 0.0006 | 0 | 827 | No | 0.9994 | 0.0006 | 0 |
| 443 | No | 0.9994 | 0.0006 | 0 | 217 | No | 0.9994 | 0.0006 | 0 |
| 309 | No | 0.9994 | 0.0006 | 0 | 929 | No | 0.9994 | 0.0006 | 0 |
| 823 | No | 0.9994 | 0.0006 | 0 | 475 | No | 0.9994 | 0.0006 | 0 |
| 961 | No | 0.9994 | 0.0006 | 0 | 783 | No | 0.9994 | 0.0006 | 0 |
| 231 | No | 0.9994 | 0.0006 | 0 | 92 | No | 0.9994 | 0.0006 | 0 |
| 862 | No | 0.9994 | 0.0006 | 0 | 491 | No | 0.9994 | 0.0006 | 0 |
| 29 | No | 0.9994 | 0.0006 | 0 | 967 | No | 0.9994 | 0.0006 | 0 |
| 222 | No | 0.9994 | 0.0006 | 0 | 623 | No | 0.9994 | 0.0006 | 0 |
| 727 | No | 0.9994 | 0.0006 | 0 | 549 | No | 0.9994 | 0.0006 | 0 |
| 149 | No | 0.9994 | 0.0006 | 0 | 780 | No | 0.9994 | 0.0006 | 0 |
| 820 | No | 0.9994 | 0.0006 | 0 | 903 | No | 0.9994 | 0.0006 | 0 |
| 917 | No | 0.9994 | 0.0006 | 0 | 645 | No | 0.9994 | 0.0006 | 0 |
| 846 | No | 0.9994 | 0.0006 | 0 | 612 | No | 0.9994 | 0.0006 | 0 |
| 162 | No | 0.9994 | 0.0006 | 0 | 526 | No | 0.9994 | 0.0006 | 0 |
| 124 | No | 0.9994 | 0.0006 | 0 | 18 | No | 0.9994 | 0.0006 | 0 |
| 671 | No | 0.9994 | 0.0006 | 0 | 962 | No | 0.9994 | 0.0006 | 0 |
| 185 | No | 0.9994 | 0.0006 | 0 | 522 | No | 0.9994 | 0.0006 | 0 |
| 682 | No | 0.9994 | 0.0006 | 0 | 345 | No | 0.9994 | 0.0006 | 0 |
| 469 | No | 0.9994 | 0.0006 | 0 | 64 | No | 0.9994 | 0.0006 | 0 |
| 367 | No | 0.9994 | 0.0006 | 0 | 183 | No | 0.9994 | 0.0006 | 0 |
| 615 | No | 0.9994 | 0.0006 | 0 | 519 | No | 0.9994 | 0.0006 | 0 |
| 584 | No | 0.9994 | 0.0006 | 0 | 401 | No | 0.9994 | 0.0006 | 0 |
| 109 | No | 0.9994 | 0.0006 | 0 | 320 | No | 0.9994 | 0.0006 | 0 |
| 73 | No | 0.9994 | 0.0006 | 0 | 91 | No | 0.9994 | 0.0006 | 0 |
| 399 | No | 0.9994 | 0.0006 | 0 | 768 | No | 0.9994 | 0.0006 | 0 |
| 14 | No | 0.9994 | 0.0006 | 0 | 141 | No | 0.9994 | 0.0006 | 0 |
| 114 | No | 0.9994 | 0.0006 | 0 | 794 | No | 0.9994 | 0.0006 | 0 |
| 486 | No | 0.9994 | 0.0006 | 0 | 356 | No | 0.9994 | 0.0006 | 0 |
| 275 | No | 0.9994 | 0.0006 | 0 | 580 | No | 0.9994 | 0.0006 | 0 |
| 810 | No | 0.9994 | 0.0006 | 0 | 16 | No | 0.9994 | 0.0006 | 0 |
| 725 | No | 0.9994 | 0.0006 | 0 | 360 | No | 0.9994 | 0.0006 | 0 |
| 778 | No | 0.9994 | 0.0006 | 0 | 687 | No | 0.9994 | 0.0006 | 0 |
| 118 | No | 0.9994 | 0.0006 | 0 | 568 | No | 0.9994 | 0.0006 | 0 |
| 412 | No | 0.9994 | 0.0006 | 0 | 483 | No | 0.9994 | 0.0006 | 0 |
| 348 | No | 0.9994 | 0.0006 | 0 | 500 | No | 0.9994 | 0.0006 | 0 |
| 735 | No | 0.9994 | 0.0006 | 0 | 566 | No | 0.9994 | 0.0006 | 0 |
| 436 | No | 0.9994 | 0.0006 | 0 | 614 | No | 0.9994 | 0.0006 | 0 |
| 152 | No | 0.9994 | 0.0006 | 0 | 204 | No | 0.9994 | 0.0006 | 0 |
| 94 | No | 0.9994 | 0.0006 | 0 | 424 | No | 0.9994 | 0.0006 | 0 |
| 636 | No | 0.9994 | 0.0006 | 0 | 445 | No | 0.9994 | 0.0006 | 0 |
| 147 | No | 0.9994 | 0.0006 | 0 | 543 | No | 0.9994 | 0.0006 | 0 |
| 196 | No | 0.9994 | 0.0006 | 0 | 433 | No | 0.9994 | 0.0006 | 0 |
| 958 | No | 0.9994 | 0.0006 | 0 | 822 | No | 0.9994 | 0.0006 | 0 |
| 478 | No | 0.9994 | 0.0006 | 0 | 326 | No | 0.9994 | 0.0006 | 0 |
| 536 | No | 0.9994 | 0.0006 | 0 | 332 | No | 0.9994 | 0.0006 | 0 |
| 933 | No | 0.9994 | 0.0006 | 0 | 126 | No | 0.9994 | 0.0006 | 0 |
| 51 | No | 0.9994 | 0.0006 | 0 | 22 | No | 0.9994 | 0.0006 | 0 |
| 191 | No | 0.9994 | 0.0006 | 0 | 868 | No | 0.9994 | 0.0006 | 0 |
| 28 | No | 0.9994 | 0.0006 | 0 | 439 | No | 0.9994 | 0.0006 | 0 |
| 38 | No | 0.9994 | 0.0006 | 0 | 80 | No | 0.9994 | 0.0006 | 0 |
| 68 | No | 0.9994 | 0.0006 | 0 | 557 | No | 0.9994 | 0.0006 | 0 |
| 176 | No | 0.9994 | 0.0006 | 0 | 27 | No | 0.9994 | 0.0006 | 0 |
| 444 | No | 0.9994 | 0.0006 | 0 | 195 | No | 0.9994 | 0.0006 | 0 |
| 650 | No | 0.9994 | 0.0006 | 0 | 954 | No | 0.9994 | 0.0006 | 0 |
| 589 | No | 0.9994 | 0.0006 | 0 | 739 | No | 0.9994 | 0.0006 | 0 |
| 870 | No | 0.9994 | 0.0006 | 0 | 956 | No | 0.9994 | 0.0006 | 0 |
| 343 | No | 0.9994 | 0.0006 | 0 | 77 | No | 0.9994 | 0.0006 | 0 |
| 616 | No | 0.9994 | 0.0006 | 0 | 872 | No | 0.9994 | 0.0006 | 0 |
| 434 | No | 0.9994 | 0.0006 | 0 | 78 | No | 0.9994 | 0.0006 | 0 |
| 292 | No | 0.9994 | 0.0006 | 0 | 885 | No | 0.9994 | 0.0006 | 0 |
| 315 | No | 0.9994 | 0.0006 | 0 | 120 | No | 0.9994 | 0.0006 | 0 |
| 694 | No | 0.9994 | 0.0006 | 0 | 853 | No | 0.9994 | 0.0006 | 0 |
| 71 | No | 0.9994 | 0.0006 | 0 | 285 | No | 0.9994 | 0.0006 | 0 |
| 802 | No | 0.9994 | 0.0006 | 0 | 376 | No | 0.9994 | 0.0006 | 0 |
| 599 | No | 0.9994 | 0.0006 | 0 | 619 | No | 0.9994 | 0.0006 | 0 |
| 236 | No | 0.9994 | 0.0006 | 0 | 782 | No | 0.9994 | 0.0006 | 0 |
| 695 | No | 0.9994 | 0.0006 | 0 | 970 | No | 0.9994 | 0.0006 | 0 |
| 626 | No | 0.9994 | 0.0006 | 0 | 897 | No | 0.9994 | 0.0006 | 0 |
| 756 | No | 0.9994 | 0.0006 | 0 | 255 | No | 0.9994 | 0.0006 | 0 |
| 460 | No | 0.9994 | 0.0006 | 0 | 188 | No | 0.9994 | 0.0006 | 0 |
| 934 | No | 0.9994 | 0.0006 | 0 | 838 | No | 0.9994 | 0.0006 | 0 |
| 710 | No | 0.9994 | 0.0006 | 0 | 298 | No | 0.9994 | 0.0006 | 0 |
| 609 | No | 0.9939 | 0.0061 | 0 | 32 | No | 0.9994 | 0.0006 | 0 |
| 936 | No | 0.9939 | 0.0061 | 0 | 575 | No | 0.9994 | 0.0006 | 0 |
| 509 | No | 0.9939 | 0.0061 | 0 | 174 | No | 0.9994 | 0.0006 | 0 |
| 81 | No | 0.9939 | 0.0061 | 0 | 24 | No | 0.9994 | 0.0006 | 0 |
| 340 | No | 0.9939 | 0.0061 | 0 | 382 | No | 0.9994 | 0.0006 | 0 |
| 926 | No | 0.9939 | 0.0061 | 0 | 759 | No | 0.9994 | 0.0006 | 0 |
| 781 | No | 0.9939 | 0.0061 | 0 | 307 | No | 0.9994 | 0.0006 | 0 |
| 844 | No | 0.9205 | 0.0795 | 0 | 643 | No | 0.9994 | 0.0006 | 0 |
| 857 | No | 0.9205 | 0.0795 | 0 | 192 | No | 0.9994 | 0.0006 | 0 |
| 608 | No | 0.9205 | 0.0795 | 0 | 731 | No | 0.9994 | 0.0006 | 0 |
| 702 | No | 0.9205 | 0.0795 | 0 | 700 | No | 0.9994 | 0.0006 | 0 |
| 646 | No | 0.9205 | 0.0795 | 0 | 334 | No | 0.9994 | 0.0006 | 0 |
| 468 | No | 0.5432 | 0.4568 | 0 | 683 | No | 0.9994 | 0.0006 | 0 |
| 155 | No | 0.9441 | 0.0559 | 0 | 254 | No | 0.9994 | 0.0006 | 0 |
| 939 | No | 0.9441 | 0.0559 | 0 | 213 | No | 0.9994 | 0.0006 | 0 |
| 221 | No | 0.9441 | 0.0559 | 0 | 53 | No | 0.9994 | 0.0006 | 0 |
| 765 | No | 0.9441 | 0.0559 | 0 | 224 | No | 0.9994 | 0.0006 | 0 |
| 265 | No | 0.9441 | 0.0559 | 0 | 834 | No | 0.9994 | 0.0006 | 0 |
| 951 | No | 0.9441 | 0.0559 | 0 | 772 | No | 0.9994 | 0.0006 | 0 |
| 569 | No | 0.9441 | 0.0559 | 0 | 30 | No | 0.9994 | 0.0006 | 0 |
| 707 | No | 0.9441 | 0.0559 | 0 | 625 | No | 0.9994 | 0.0006 | 0 |
| 212 | No | 0.9441 | 0.0559 | 0 | 901 | No | 0.9994 | 0.0006 | 0 |
| 848 | No | 0.9441 | 0.0559 | 0 | 642 | No | 0.9994 | 0.0006 | 0 |
| 831 | No | 0.9441 | 0.0559 | 0 | 720 | No | 0.9994 | 0.0006 | 0 |
| 455 | No | 0.9441 | 0.0559 | 0 | 688 | No | 0.9994 | 0.0006 | 0 |
| 666 | No | 0.9441 | 0.0559 | 0 | 649 | No | 0.9994 | 0.0006 | 0 |
| 1 | No | 0.9441 | 0.0559 | 0 | 157 | No | 0.9994 | 0.0006 | 0 |
| 264 | No | 0.9441 | 0.0559 | 0 | 906 | No | 0.9994 | 0.0006 | 0 |
| 899 | No | 0.9441 | 0.0559 | 0 | 837 | No | 0.9994 | 0.0006 | 0 |
| 441 | No | 0.9441 | 0.0559 | 0 | 240 | No | 0.9994 | 0.0006 | 0 |
| 295 | No | 0.9441 | 0.0559 | 0 | 449 | No | 0.9994 | 0.0006 | 0 |
| 214 | No | 0.9441 | 0.0559 | 0 | 238 | No | 0.9994 | 0.0006 | 0 |
| 19 | No | 0.9441 | 0.0559 | 0 | 640 | No | 0.9994 | 0.0006 | 0 |
| 55 | No | 0.9441 | 0.0559 | 0 | 418 | No | 0.9994 | 0.0006 | 0 |
| 372 | No | 0.9441 | 0.0559 | 0 | 633 | No | 0.9994 | 0.0006 | 0 |
| 621 | No | 0.9441 | 0.0559 | 0 | 770 | No | 0.9994 | 0.0006 | 0 |
| 590 | No | 0.9441 | 0.0559 | 0 | 448 | No | 0.9994 | 0.0006 | 0 |
| 383 | No | 0.9441 | 0.0559 | 0 | 229 | No | 0.9994 | 0.0006 | 0 |
| 323 | No | 0.9441 | 0.0559 | 0 | 512 | No | 0.9994 | 0.0006 | 0 |
| 435 | No | 0.9441 | 0.0559 | 0 | 527 | No | 0.9994 | 0.0006 | 0 |
| 247 | No | 0.9441 | 0.0559 | 0 | 813 | No | 0.9994 | 0.0006 | 0 |
| 555 | No | 0.9441 | 0.0559 | 0 | 3 | No | 0.9994 | 0.0006 | 0 |
| 696 | No | 0.9441 | 0.0559 | 0 | 490 | No | 0.9994 | 0.0006 | 0 |
| 223 | No | 0.9441 | 0.0559 | 0 | 538 | No | 0.9994 | 0.0006 | 0 |
| 172 | No | 0.9441 | 0.0559 | 0 | 921 | No | 0.9994 | 0.0006 | 0 |
| 245 | No | 0.9441 | 0.0559 | 0 | 408 | No | 0.9994 | 0.0006 | 0 |
| 713 | No | 0.9441 | 0.0559 | 0 | 665 | No | 0.9994 | 0.0006 | 0 |
| 570 | No | 0.9441 | 0.0559 | 0 | 165 | No | 0.9994 | 0.0006 | 0 |
| 447 | No | 0.9441 | 0.0559 | 0 | 593 | No | 0.9994 | 0.0006 | 0 |
| 732 | No | 0.9441 | 0.0559 | 0 | 734 | No | 0.9994 | 0.0006 | 0 |
| 843 | No | 0.9441 | 0.0559 | 0 | 803 | No | 0.9994 | 0.0006 | 0 |
| 244 | No | 0.9441 | 0.0559 | 0 | 891 | No | 0.9994 | 0.0006 | 0 |
| 832 | No | 0.9441 | 0.0559 | 0 | 179 | No | 0.9994 | 0.0006 | 0 |
| 397 | No | 0.9441 | 0.0559 | 0 | 552 | No | 0.9994 | 0.0006 | 0 |
| 48 | No | 0.9441 | 0.0559 | 0 | 859 | No | 0.9994 | 0.0006 | 0 |
| 54 | No | 0.9441 | 0.0559 | 0 | 689 | No | 0.9994 | 0.0006 | 0 |
| 841 | No | 0.9441 | 0.0559 | 0 | 411 | No | 0.9994 | 0.0006 | 0 |
| 59 | No | 0.9441 | 0.0559 | 0 | 337 | No | 0.9994 | 0.0006 | 0 |
| 426 | No | 0.9441 | 0.0559 | 0 | 473 | No | 0.9994 | 0.0006 | 0 |
| 163 | No | 0.9441 | 0.0559 | 0 | 867 | No | 0.9994 | 0.0006 | 0 |
| 654 | No | 0.9441 | 0.0559 | 0 | 940 | No | 0.9994 | 0.0006 | 0 |
| 467 | No | 0.9441 | 0.0559 | 0 | 421 | No | 0.9994 | 0.0006 | 0 |
| 560 | No | 0.6341 | 0.3659 | 0 | 153 | No | 0.9994 | 0.0006 | 0 |
| 58 | No | 0.6341 | 0.3659 | 0 | 728 | No | 0.9994 | 0.0006 | 0 |
| 474 | No | 0.1104 | 0.8896 | 0 | 430 | No | 0.9994 | 0.0006 | 0 |
| 253 | No | 0.1104 | 0.8896 | 0 | 883 | No | 0.9994 | 0.0006 | 0 |
| 881 | No | 0.1104 | 0.8896 | 0 | 674 | No | 0.9994 | 0.0006 | 0 |
| 639 | No | 0.1104 | 0.8896 | 0 | 263 | No | 0.9939 | 0.0061 | 0 |
| 775 | No | 0.1104 | 0.8896 | 0 | 122 | No | 0.9939 | 0.0061 | 0 |
| 588 | No | 0.1104 | 0.8896 | 0 | 709 | No | 0.9939 | 0.0061 | 0 |
| 273 | No | 0.1104 | 0.8896 | 0 | 477 | No | 0.9939 | 0.0061 | 0 |
| 336 | No | 0.1104 | 0.8896 | 0 | 79 | No | 0.9208 | 0.0792 | 0 |
| 677 | No | 0.1104 | 0.8896 | 0 | 495 | No | 0.9208 | 0.0792 | 0 |
| 747 | No | 0.1104 | 0.8896 | 0 | 139 | No | 0.9208 | 0.0792 | 0 |
| 306 | No | 0.1104 | 0.8896 | 0 | 669 | No | 0.9208 | 0.0792 | 0 |
| 452 | No | 0.1104 | 0.8896 | 0 | 166 | No | 0.9208 | 0.0792 | 0 |
| 605 | No | 0.1104 | 0.8896 | 0 | 432 | No | 0.9443 | 0.0557 | 0 |
| 784 | No | 0.1104 | 0.8896 | 0 | 622 | No | 0.9443 | 0.0557 | 0 |
| 227 | No | 0.1104 | 0.8896 | 0 | 684 | No | 0.9443 | 0.0557 | 0 |
| 828 | No | 0.1104 | 0.8896 | 0 | 721 | No | 0.9443 | 0.0557 | 0 |
| 595 | No | 0.1104 | 0.8896 | 0 | 331 | No | 0.9443 | 0.0557 | 0 |
| 400 | No | 0.1104 | 0.8896 | 0 | 554 | No | 0.9443 | 0.0557 | 0 |
| 826 | No | 0.0126 | 0.9874 | 0 | 574 | No | 0.9443 | 0.0557 | 0 |
| 237 | No | 0.9949 | 0.0051 | 0 | 610 | No | 0.9443 | 0.0557 | 0 |
| 592 | No | 0.9949 | 0.0051 | 0 | 243 | No | 0.9443 | 0.0557 | 0 |
| 737 | No | 0.9949 | 0.0051 | 0 | 914 | No | 0.9443 | 0.0557 | 0 |
| 964 | No | 0.9949 | 0.0051 | 0 | 919 | No | 0.9443 | 0.0557 | 0 |
| 66 | No | 0.9949 | 0.0051 | 0 | 745 | No | 0.9443 | 0.0557 | 0 |
| 712 | No | 0.9949 | 0.0051 | 0 | 947 | No | 0.9443 | 0.0557 | 0 |
| 329 | No | 0.9949 | 0.0051 | 0 | 955 | No | 0.9443 | 0.0557 | 0 |
| 100 | No | 0.9949 | 0.0051 | 0 | 767 | No | 0.9443 | 0.0557 | 0 |
| 206 | No | 0.9949 | 0.0051 | 0 | 93 | No | 0.9443 | 0.0557 | 0 |
| 131 | No | 0.9949 | 0.0051 | 0 | 304 | No | 0.9443 | 0.0557 | 0 |
| 248 | No | 0.9949 | 0.0051 | 0 | 50 | No | 0.9443 | 0.0557 | 0 |
| 551 | No | 0.9949 | 0.0051 | 0 | 305 | No | 0.9443 | 0.0557 | 0 |
| 113 | No | 0.9949 | 0.0051 | 0 | 600 | No | 0.9443 | 0.0557 | 0 |
| 197 | No | 0.9949 | 0.0051 | 0 | 850 | No | 0.9443 | 0.0557 | 0 |
| 505 | No | 0.9949 | 0.0051 | 0 | 556 | No | 0.9443 | 0.0557 | 0 |
| 463 | No | 0.9949 | 0.0051 | 0 | 528 | No | 0.9443 | 0.0557 | 0 |
| 662 | No | 0.9949 | 0.0051 | 0 | 738 | No | 0.9443 | 0.0557 | 0 |
| 711 | No | 0.9949 | 0.0051 | 0 | 753 | No | 0.9443 | 0.0557 | 0 |
| 301 | No | 0.9949 | 0.0051 | 0 | 350 | No | 0.9443 | 0.0557 | 0 |
| 816 | No | 0.9949 | 0.0051 | 0 | 466 | No | 0.9443 | 0.0557 | 0 |
| 225 | No | 0.9949 | 0.0051 | 0 | 429 | No | 0.9443 | 0.0557 | 0 |
| 31 | No | 0.9949 | 0.0051 | 0 | 851 | No | 0.9443 | 0.0557 | 0 |
| 880 | No | 0.9949 | 0.0051 | 0 | 847 | No | 0.9443 | 0.0557 | 0 |
| 498 | No | 0.9949 | 0.0051 | 0 | 740 | No | 0.9443 | 0.0557 | 0 |
| 655 | No | 0.9524 | 0.0476 | 0 | 960 | No | 0.9443 | 0.0557 | 0 |
| 274 | No | 0.9524 | 0.0476 | 0 | 413 | No | 0.9443 | 0.0557 | 0 |
| 351 | No | 0.5887 | 0.4113 | 0 | 913 | No | 0.9443 | 0.0557 | 0 |
| 637 | No | 0.676 | 0.324 | 0 | 681 | No | 0.9443 | 0.0557 | 0 |
| 748 | No | 0.676 | 0.324 | 0 | 111 | No | 0.9443 | 0.0557 | 0 |
| 417 | No | 0.676 | 0.324 | 0 | 84 | No | 0.9443 | 0.0557 | 0 |
| 123 | No | 0.676 | 0.324 | 0 | 299 | No | 0.9443 | 0.0557 | 0 |
| 686 | No | 0.676 | 0.324 | 0 | 907 | No | 0.9443 | 0.0557 | 0 |
| 233 | No | 0.676 | 0.324 | 0 | 112 | No | 0.9443 | 0.0557 | 0 |
| 398 | No | 0.676 | 0.324 | 0 | 743 | No | 0.9443 | 0.0557 | 0 |
| 931 | No | 0.676 | 0.324 | 0 | 548 | No | 0.9443 | 0.0557 | 0 |
| 258 | No | 0.676 | 0.324 | 0 | 858 | No | 0.9443 | 0.0557 | 0 |
| 817 | No | 0.676 | 0.324 | 0 | 668 | No | 0.9443 | 0.0557 | 0 |
| 927 | No | 0.676 | 0.324 | 0 | 8 | No | 0.635 | 0.365 | 0 |
| 290 | No | 0.676 | 0.324 | 0 | 758 | No | 0.635 | 0.365 | 0 |
| 534 | No | 0.676 | 0.324 | 0 | 715 | No | 0.635 | 0.365 | 0 |
| 496 | No | 0.676 | 0.324 | 0 | 741 | No | 0.1107 | 0.8893 | 0 |
| 849 | No | 0.676 | 0.324 | 0 | 541 | No | 0.1107 | 0.8893 | 0 |
| 20 | No | 0.676 | 0.324 | 0 | 7 | No | 0.1107 | 0.8893 | 0 |
| 391 | No | 0.676 | 0.324 | 0 | 310 | No | 0.1107 | 0.8893 | 0 |
| 302 | No | 0.676 | 0.324 | 0 | 520 | No | 0.1107 | 0.8893 | 0 |
| 291 | No | 0.676 | 0.324 | 0 | 497 | No | 0.1107 | 0.8893 | 0 |
| 480 | No | 0.1764 | 0.8236 | 0 | 365 | No | 0.1107 | 0.8893 | 0 |
| 798 | No | 0.1764 | 0.8236 | 0 | 876 | No | 0.1107 | 0.8893 | 0 |
| 388 | No | 0.1764 | 0.8236 | 0 | 656 | No | 0.1107 | 0.8893 | 0 |
| 287 | No | 0.1764 | 0.8236 | 0 | 414 | No | 0.1107 | 0.8893 | 0 |
| 377 | No | 0.1764 | 0.8236 | 0 | 39 | No | 0.1107 | 0.8893 | 0 |
| 104 | No | 0.1764 | 0.8236 | 0 | 215 | No | 0.1107 | 0.8893 | 0 |
| 703 | No | 0.0151 | 0.9849 | 0 | 755 | No | 0.1107 | 0.8893 | 0 |
| 199 | No | 0.0151 | 0.9849 | 0 | 23 | No | 0.1107 | 0.8893 | 0 |
| 97 | No | 0.0151 | 0.9849 | 0 | 499 | No | 0.1107 | 0.8893 | 0 |
| 381 | No | 0.0151 | 0.9849 | 0 | 660 | No | 0.1107 | 0.8893 | 0 |
| 261 | No | 0.0151 | 0.9849 | 0 | 47 | No | 0.1107 | 0.8893 | 0 |
| 788 | No | 0.0151 | 0.9849 | 0 | 506 | No | 0.1107 | 0.8893 | 0 |
| 773 | No | 0.0151 | 0.9849 | 0 | 208 | No | 0.1107 | 0.8893 | 0 |
| 395 | No | 0.0151 | 0.9849 | 0 | 357 | No | 0.0126 | 0.9874 | 0 |
| 98 | No | 0.0151 | 0.9849 | 0 | 262 | No | 0.0126 | 0.9874 | 0 |
| 419 | No | 0.0151 | 0.9849 | 0 | 406 | No | 0.0126 | 0.9874 | 0 |
| 511 | No | 0.0151 | 0.9849 | 0 | 232 | No | 0.0126 | 0.9874 | 0 |
| 730 | No | 0.0151 | 0.9849 | 0 | 630 | No | 0.0126 | 0.9874 | 0 |
| 321 | No | 0.0151 | 0.9849 | 0 | 631 | No | 0.0126 | 0.9874 | 0 |
| 815 | No | 0.0151 | 0.9849 | 0 | 953 | No | 0.9949 | 0.0051 | 0 |
| 799 | No | 0.0151 | 0.9849 | 0 | 220 | No | 0.9949 | 0.0051 | 0 |
| 482 | No | 0.0151 | 0.9849 | 0 | 277 | No | 0.9949 | 0.0051 | 0 |
| 161 | No | 0.0151 | 0.9849 | 0 | 910 | No | 0.9949 | 0.0051 | 0 |
| 807 | No | 0.0151 | 0.9849 | 0 | 35 | No | 0.9949 | 0.0051 | 0 |
| 800 | No | 0.0151 | 0.9849 | 0 | 330 | No | 0.9949 | 0.0051 | 0 |
| 487 | No | 0.0151 | 0.9849 | 0 | 749 | No | 0.9949 | 0.0051 | 0 |
| 821 | No | 0.0151 | 0.9849 | 0 | 472 | No | 0.9949 | 0.0051 | 0 |
| 811 | No | 0.0151 | 0.9849 | 0 | 529 | No | 0.9949 | 0.0051 | 0 |
| 207 | No | 0.0151 | 0.9849 | 0 | 143 | No | 0.9949 | 0.0051 | 0 |
| 692 | No | 0.0151 | 0.9849 | 0 | 685 | No | 0.9949 | 0.0051 | 0 |
| 825 | No | 0.0151 | 0.9849 | 0 | 312 | No | 0.9949 | 0.0051 | 0 |
| 311 | No | 0.0151 | 0.9849 | 0 | 279 | No | 0.9949 | 0.0051 | 0 |
| 648 | No | 0.0151 | 0.9849 | 0 | 932 | No | 0.9949 | 0.0051 | 0 |
| 535 | No | 0.0151 | 0.9849 | 0 | 744 | No | 0.9949 | 0.0051 | 0 |
| 82 | No | 0.0151 | 0.9849 | 0 | 829 | No | 0.9949 | 0.0051 | 0 |
| 335 | No | 0.0151 | 0.9849 | 0 | 370 | No | 0.9949 | 0.0051 | 0 |
| 453 | No | 0.0151 | 0.9849 | 0 | 545 | No | 0.9949 | 0.0051 | 0 |
| 353 | No | 0.0151 | 0.9849 | 0 | 270 | No | 0.9949 | 0.0051 | 0 |
| 169 | No | 0.0016 | 0.9984 | 0 | 160 | No | 0.9949 | 0.0051 | 0 |
| 90 | No | 0.0016 | 0.9984 | 0 | 761 | No | 0.9949 | 0.0051 | 0 |
| 587 | No | 0.0016 | 0.9984 | 0 | 544 | No | 0.9949 | 0.0051 | 0 |
| 280 | No | 0.0016 | 0.9984 | 0 | 617 | No | 0.9949 | 0.0051 | 0 |
| 145 | No | 0.0016 | 0.9984 | 0 | 546 | No | 0.9949 | 0.0051 | 0 |
| 886 | No | 0.0016 | 0.9984 | 0 | 167 | No | 0.9949 | 0.0051 | 0 |
| 596 | No | 0.0016 | 0.9984 | 0 | 62 | No | 0.9949 | 0.0051 | 0 |
| 328 | No | 0.0016 | 0.9984 | 0 | 115 | No | 0.9949 | 0.0051 | 0 |
| 297 | No | 0.0016 | 0.9984 | 0 | 128 | No | 0.9949 | 0.0051 | 0 |
| 819 | No | 0.0016 | 0.9984 | 0 | 159 | No | 0.9949 | 0.0051 | 0 |
| 501 | No | 0.0016 | 0.9984 | 0 | 481 | No | 0.9949 | 0.0051 | 0 |
| 385 | No | 0.0016 | 0.9984 | 0 | 470 | No | 0.9949 | 0.0051 | 0 |
| 705 | No | 0.0016 | 0.9984 | 0 | 150 | No | 0.9949 | 0.0051 | 0 |
| 40 | No | 0.0016 | 0.9984 | 0 | 902 | No | 0.9949 | 0.0051 | 0 |
| 364 | newly | 0.9988 | 0.0012 | 0 | 601 | No | 0.9949 | 0.0051 | 0 |
| 200 | newly | 0.9988 | 0.0012 | 0 | 303 | No | 0.9949 | 0.0051 | 0 |
| 628 | newly | 0.9988 | 0.0012 | 0 | 457 | No | 0.9949 | 0.0051 | 0 |
| 895 | newly | 0.9988 | 0.0012 | 0 | 484 | No | 0.9949 | 0.0051 | 0 |
| 83 | newly | 0.9988 | 0.0012 | 0 | 723 | No | 0.9949 | 0.0051 | 0 |
| 6 | newly | 0.9988 | 0.0012 | 0 | 909 | No | 0.9949 | 0.0051 | 0 |
| 708 | newly | 0.9988 | 0.0012 | 0 | 316 | No | 0.9949 | 0.0051 | 0 |
| 888 | newly | 0.9988 | 0.0012 | 0 | 900 | No | 0.9949 | 0.0051 | 0 |
| 338 | newly | 0.9988 | 0.0012 | 0 | 852 | No | 0.9949 | 0.0051 | 0 |
| 363 | newly | 0.9988 | 0.0012 | 0 | 806 | No | 0.9949 | 0.0051 | 0 |
| 85 | newly | 0.9988 | 0.0012 | 0 | 503 | No | 0.9949 | 0.0051 | 0 |
| 923 | newly | 0.9988 | 0.0012 | 0 | 44 | No | 0.9949 | 0.0051 | 0 |
| 583 | newly | 0.9988 | 0.0012 | 0 | 266 | No | 0.9949 | 0.0051 | 0 |
| 471 | newly | 0.9988 | 0.0012 | 0 | 211 | No | 0.9949 | 0.0051 | 0 |
| 701 | newly | 0.9988 | 0.0012 | 0 | 476 | No | 0.9949 | 0.0051 | 0 |
| 746 | newly | 0.9988 | 0.0012 | 0 | 559 | No | 0.9949 | 0.0051 | 0 |
| 431 | newly | 0.9988 | 0.0012 | 0 | 380 | No | 0.9949 | 0.0051 | 0 |
| 409 | newly | 0.9988 | 0.0012 | 0 | 752 | No | 0.9949 | 0.0051 | 0 |
| 581 | newly | 0.9988 | 0.0012 | 0 | 854 | No | 0.9949 | 0.0051 | 0 |
| 230 | newly | 0.9988 | 0.0012 | 0 | 606 | No | 0.9949 | 0.0051 | 0 |
| 96 | newly | 0.9988 | 0.0012 | 0 | 918 | No | 0.9949 | 0.0051 | 0 |
| 539 | newly | 0.9988 | 0.0012 | 0 | 4 | No | 0.9949 | 0.0051 | 0 |
| 375 | newly | 0.9988 | 0.0012 | 0 | 210 | No | 0.9949 | 0.0051 | 0 |
| 89 | newly | 0.9988 | 0.0012 | 0 | 618 | No | 0.9949 | 0.0051 | 0 |
| 577 | newly | 0.9988 | 0.0012 | 0 | 576 | No | 0.9949 | 0.0051 | 0 |
| 402 | newly | 0.9988 | 0.0012 | 0 | 69 | No | 0.9949 | 0.0051 | 0 |
| 465 | newly | 0.9988 | 0.0012 | 0 | 281 | No | 0.9949 | 0.0051 | 0 |
| 771 | newly | 0.9988 | 0.0012 | 0 | 532 | No | 0.9949 | 0.0051 | 0 |
| 125 | newly | 0.9988 | 0.0012 | 0 | 135 | No | 0.9949 | 0.0051 | 0 |
| 317 | newly | 0.9988 | 0.0012 | 0 | 818 | No | 0.9949 | 0.0051 | 0 |
| 425 | newly | 0.9988 | 0.0012 | 0 | 186 | No | 0.9949 | 0.0051 | 0 |
| 523 | newly | 0.9988 | 0.0012 | 0 | 374 | No | 0.9949 | 0.0051 | 0 |
| 757 | newly | 0.9988 | 0.0012 | 0 | 164 | No | 0.9949 | 0.0051 | 0 |
| 896 | newly | 0.9988 | 0.0012 | 0 | 691 | No | 0.9526 | 0.0474 | 0 |
| 36 | newly | 0.9988 | 0.0012 | 0 | 182 | No | 0.9526 | 0.0474 | 0 |
| 718 | newly | 0.9988 | 0.0012 | 0 | 278 | No | 0.5897 | 0.4103 | 0 |
| 404 | newly | 0.9988 | 0.0012 | 0 | 347 | No | 0.5897 | 0.4103 | 0 |
| 193 | newly | 0.9988 | 0.0012 | 0 | 272 | No | 0.5897 | 0.4103 | 0 |
| 699 | newly | 0.9884 | 0.0116 | 0 | 2 | No | 0.5897 | 0.4103 | 0 |
| 133 | newly | 0.9884 | 0.0116 | 0 | 915 | No | 0.1285 | 0.8715 | 0 |
| 553 | newly | 0.8595 | 0.1405 | 0 | 594 | No | 0.6768 | 0.3232 | 0 |
| 184 | newly | 0.8992 | 0.1008 | 0 | 922 | No | 0.6768 | 0.3232 | 0 |
| 319 | newly | 0.8992 | 0.1008 | 0 | 935 | No | 0.6768 | 0.3232 | 0 |
| 836 | newly | 0.8992 | 0.1008 | 0 | 389 | No | 0.6768 | 0.3232 | 0 |
| 41 | newly | 0.8992 | 0.1008 | 0 | 916 | No | 0.6768 | 0.3232 | 0 |
| 25 | newly | 0.8992 | 0.1008 | 0 | 349 | No | 0.6768 | 0.3232 | 0 |
| 808 | newly | 0.8992 | 0.1008 | 0 | 420 | No | 0.6768 | 0.3232 | 0 |
| 458 | newly | 0.8992 | 0.1008 | 0 | 944 | No | 0.6768 | 0.3232 | 0 |
| 874 | newly | 0.4779 | 0.5221 | 0 | 11 | No | 0.6768 | 0.3232 | 0 |
| 252 | newly | 0.0615 | 0.9385 | 0 | 824 | No | 0.6768 | 0.3232 | 0 |
| 479 | newly | 0.0615 | 0.9385 | 0 | 809 | No | 0.6768 | 0.3232 | 0 |
| 205 | newly | 0.0615 | 0.9385 | 0 | 892 | No | 0.6768 | 0.3232 | 0 |
| 865 | newly | 0.0615 | 0.9385 | 0 | 884 | No | 0.6768 | 0.3232 | 0 |
| 344 | newly | 0.0615 | 0.9385 | 0 | 789 | No | 0.6768 | 0.3232 | 0 |
| 952 | newly | 0.9904 | 0.0096 | 0 | 300 | No | 0.6768 | 0.3232 | 0 |
| 144 | newly | 0.9135 | 0.0865 | 0 | 289 | No | 0.6768 | 0.3232 | 0 |
| 864 | newly | 0.9135 | 0.0865 | 0 | 598 | No | 0.6768 | 0.3232 | 0 |
| 288 | newly | 0.9135 | 0.0865 | 0 | 52 | No | 0.6768 | 0.3232 | 0 |
| 845 | newly | 0.4305 | 0.5695 | 0 | 15 | No | 0.1769 | 0.8231 | 0 |
| 119 | newly | 0.072 | 0.928 | 0 | 354 | No | 0.0152 | 0.9848 | 0 |
| 156 | newly | 0.5242 | 0.4758 | 0 | 791 | No | 0.0152 | 0.9848 | 0 |
| 774 | newly | 0.5242 | 0.4758 | 0 | 485 | No | 0.0152 | 0.9848 | 0 |
| 359 | newly | 0.5242 | 0.4758 | 0 | 63 | No | 0.0152 | 0.9848 | 0 |
| 515 | newly | 0.5242 | 0.4758 | 0 | 941 | No | 0.0152 | 0.9848 | 0 |
| 742 | newly | 0.1016 | 0.8984 | 0 | 142 | No | 0.0152 | 0.9848 | 0 |
| 839 | newly | 0.1016 | 0.8984 | 0 | 386 | No | 0.0152 | 0.9848 | 0 |
| 110 | newly | 0.1016 | 0.8984 | 0 | 680 | No | 0.0152 | 0.9848 | 0 |
| 65 | newly | 0.1016 | 0.8984 | 0 | 407 | No | 0.0152 | 0.9848 | 0 |
| 786 | newly | 0.008 | 0.992 | 0 | 138 | No | 0.0152 | 0.9848 | 0 |
| 693 | newly | 0.008 | 0.992 | 0 | 416 | No | 0.0152 | 0.9848 | 0 |
| 341 | newly | 0.008 | 0.992 | 0 | 116 | No | 0.0152 | 0.9848 | 0 |
| 355 | newly | 0.008 | 0.992 | 0 | 904 | No | 0.0152 | 0.9848 | 0 |
| 390 | newly | 0.008 | 0.992 | 0 | 620 | No | 0.0152 | 0.9848 | 0 |
| 657 | newly | 0.008 | 0.992 | 0 | 216 | No | 0.0152 | 0.9848 | 0 |
| 716 | newly | 0.008 | 0.992 | 0 | 578 | No | 0.0152 | 0.9848 | 0 |
| 542 | newly | 0.008 | 0.992 | 0 | 95 | No | 0.0152 | 0.9848 | 0 |
| 121 | newly | 0.008 | 0.992 | 0 | 101 | No | 0.0152 | 0.9848 | 0 |
| 804 | newly | 0.008 | 0.992 | 0 | 533 | No | 0.0152 | 0.9848 | 0 |
| 667 | newly | 0.008 | 0.992 | 0 | 72 | No | 0.0152 | 0.9848 | 0 |
| 873 | newly | 0.008 | 0.992 | 0 | 943 | No | 0.0152 | 0.9848 | 0 |
| 371 | newly | 0.0008 | 0.9992 | 0 | 567 | No | 0.0152 | 0.9848 | 0 |
| 860 | newly | 0.0008 | 0.9992 | 0 | 415 | No | 0.0152 | 0.9848 | 0 |
| 518 | newly | 0.0008 | 0.9992 | 0 | 194 | No | 0.0152 | 0.9848 | 0 |
| 750 | newly | 0.0008 | 0.9992 | 0 | 369 | No | 0.0152 | 0.9848 | 0 |
| 565 | newly | 0.0008 | 0.9992 | 0 | 464 | No | 0.0152 | 0.9848 | 0 |
| 663 | newly | 0.0008 | 0.9992 | 0 | 427 | No | 0.0016 | 0.9984 | 0 |
| 450 | newly | 0.0008 | 0.9992 | 0 | 327 | No | 0.0016 | 0.9984 | 0 |
| 260 | No | 0.9994 | 0.0006 | 0 | 21 | No | 0.0016 | 0.9984 | 0 |
| 795 | No | 0.9994 | 0.0006 | 0 | 787 | No | 0.0016 | 0.9984 | 0 |
| 99 | No | 0.9994 | 0.0006 | 0 | 673 | No | 0.0016 | 0.9984 | 0 |
| 130 | No | 0.9994 | 0.0006 | 0 | 658 | No | 0.0016 | 0.9984 | 0 |
| 751 | No | 0.9994 | 0.0006 | 0 | 890 | No | 0.0016 | 0.9984 | 0 |
| 792 | No | 0.9994 | 0.0006 | 0 | 875 | No | 0.0016 | 0.9984 | 0 |
| 879 | No | 0.9994 | 0.0006 | 0 | 379 | No | 0.0016 | 0.9984 | 0 |
| 959 | No | 0.9994 | 0.0006 | 0 | 107 | No | 0.0016 | 0.9984 | 0 |
| 190 | No | 0.9994 | 0.0006 | 0 | 840 | No | 0.0016 | 0.9984 | 0 |
| 235 | No | 0.9994 | 0.0006 | 0 | 46 | No | 0.0016 | 0.9984 | 0 |
| 342 | No | 0.9994 | 0.0006 | 0 | 203 | No | 0.0016 | 0.9984 | 0 |
| 271 | No | 0.9994 | 0.0006 | 0 | 550 | No | 0.0016 | 0.9984 | 0 |
| 508 | No | 0.9994 | 0.0006 | 0 | 530 | No | 0.0016 | 0.9984 | 0 |
| 12 | No | 0.9941 | 0.0059 | 0 | 950 | No | 0.0016 | 0.9984 | 0 |
| 117 | No | 0.9231 | 0.0769 | 0 | 729 | No | 0.0016 | 0.9984 | 0 |
| 366 | No | 0.9459 | 0.0541 | 0 | 56 | No | 0.0016 | 0.9984 | 0 |
| 324 | No | 0.9459 | 0.0541 | 0 | 507 | No | 0.0016 | 0.9984 | 0 |
| 103 | No | 0.9459 | 0.0541 | 0 | 855 | No | 0.0016 | 0.9984 | 0 |
| 234 | No | 0.6422 | 0.3578 | 0 | 403 | No | 0.0016 | 0.9984 | 0 |
| 102 | No | 0.6422 | 0.3578 | 0 | 717 | No | 0.0016 | 0.9984 | 0 |
| 762 | No | 0.1139 | 0.8861 | 0 | 604 | No | 0.0016 | 0.9984 | 0 |
| 812 | No | 0.9951 | 0.0049 | 0 | 777 | No | 0.0016 | 0.9984 | 0 |
| 670 | No | 0.9539 | 0.0461 | 0 | 108 | newly | 0.7677 | 0.0009 | 0.2313 |
| 178 | No | 0.9539 | 0.0461 | 0 | 726 | newly | 0.7677 | 0.0009 | 0.2313 |
| 629 | No | 0.6836 | 0.3164 | 0 | 562 | newly | 0.7677 | 0.0009 | 0.2313 |
| 521 | No | 0.6836 | 0.3164 | 0 | 42 | newly | 0.7677 | 0.0009 | 0.2313 |
| 524 | No | 0.6836 | 0.3164 | 0 | 189 | newly | 0.7677 | 0.0009 | 0.2313 |
| 963 | No | 0.0156 | 0.9844 | 0 | 282 | newly | 0.7677 | 0.0009 | 0.2313 |
| 456 | No | 0.0156 | 0.9844 | 0 | 202 | newly | 0.7677 | 0.0009 | 0.2313 |
| 561 | No | 0.0016 | 0.9984 | 0 | 513 | newly | 0.7677 | 0.0009 | 0.2313 |
| 572 | No | 0.0016 | 0.9984 | 0 | 313 | newly | 0.7677 | 0.0009 | 0.2313 |
| 925 | newly | 0.2745 | 0.0003 | 0.7252 | 504 | newly | 0.7677 | 0.0009 | 0.2313 |
| 146 | newly | 0.2745 | 0.0003 | 0.7252 | 603 | newly | 0.7677 | 0.0009 | 0.2313 |
| 129 | newly | 0.2745 | 0.0003 | 0.7252 | 651 | newly | 0.7677 | 0.0009 | 0.2313 |
| 659 | newly | 0.2745 | 0.0003 | 0.7252 | 833 | newly | 0.7677 | 0.0009 | 0.2313 |
| 494 | newly | 0.2745 | 0.0003 | 0.7252 | 362 | newly | 0.7677 | 0.0009 | 0.2313 |
| 898 | newly | 0.2745 | 0.0003 | 0.7252 | 17 | newly | 0.7677 | 0.0009 | 0.2313 |
| 136 | newly | 0.2745 | 0.0003 | 0.7252 | 769 | newly | 0.7677 | 0.0009 | 0.2313 |
| 635 | newly | 0.2745 | 0.0003 | 0.7252 | 573 | newly | 0.7677 | 0.0009 | 0.2313 |
| 547 | newly | 0.2745 | 0.0003 | 0.7252 | 510 | newly | 0.4985 | 0.0557 | 0.4458 |
| 308 | newly | 0.2745 | 0.0003 | 0.7252 | 492 | newly | 0.4985 | 0.0557 | 0.4458 |
| 965 | newly | 0.2745 | 0.0003 | 0.7252 | 540 | newly | 0.4985 | 0.0557 | 0.4458 |
| 525 | newly | 0.2745 | 0.0003 | 0.7252 | 613 | newly | 0.4985 | 0.0557 | 0.4458 |
| 181 | newly | 0.2745 | 0.0003 | 0.7252 | 564 | newly | 0.4985 | 0.0557 | 0.4458 |
| 571 | newly | 0.2745 | 0.0003 | 0.7252 | 173 | newly | 0.4985 | 0.0557 | 0.4458 |
| 373 | newly | 0.2745 | 0.0003 | 0.7252 | 127 | newly | 0.4985 | 0.0557 | 0.4458 |
| 318 | newly | 0.2745 | 0.0003 | 0.7252 | 405 | newly | 0.4985 | 0.0557 | 0.4458 |
| 945 | newly | 0.0335 | 0.0053 | 0.9612 | 579 | newly | 0.4985 | 0.0557 | 0.4458 |
| 76 | newly | 0.0335 | 0.0053 | 0.9612 | 428 | newly | 0.4985 | 0.0557 | 0.4458 |
| 585 | newly | 0.0335 | 0.0053 | 0.9612 | 793 | newly | 0.4789 | 0.5211 | 0 |
| 177 | newly | 0.0335 | 0.0053 | 0.9612 | 250 | newly | 0.0386 | 0.5868 | 0.3746 |
| 384 | newly | 0.1117 | 0.0121 | 0.8762 | 314 | newly | 0.0386 | 0.5868 | 0.3746 |
| 638 | newly | 0.1117 | 0.0121 | 0.8762 | 948 | newly | 0.0386 | 0.5868 | 0.3746 |
| 602 | newly | 0.1117 | 0.0121 | 0.8762 | 218 | newly | 0.0386 | 0.5868 | 0.3746 |
| 764 | newly | 0.1117 | 0.0121 | 0.8762 | 586 | newly | 0.0386 | 0.5868 | 0.3746 |
| 396 | newly | 0.1117 | 0.0121 | 0.8762 | 134 | newly | 0.0386 | 0.5868 | 0.3746 |
| 672 | newly | 0.1117 | 0.0121 | 0.8762 | 106 | newly | 0.9393 | 0.0091 | 0.0516 |
| 187 | newly | 0.1117 | 0.0121 | 0.8762 | 908 | newly | 0.9393 | 0.0091 | 0.0516 |
| 627 | newly | 0.1117 | 0.0121 | 0.8762 | 459 | newly | 0.9393 | 0.0091 | 0.0516 |
| 294 | newly | 0.0099 | 0.1462 | 0.8439 | 957 | newly | 0.9393 | 0.0091 | 0.0516 |
| 60 | newly | 0.0099 | 0.1462 | 0.8439 | 209 | newly | 0.9393 | 0.0091 | 0.0516 |
| 283 | newly | 0.0099 | 0.1462 | 0.8439 | 13 | newly | 0.9393 | 0.0091 | 0.0516 |
| 10 | newly | 0.0099 | 0.1462 | 0.8439 | 422 | newly | 0.4838 | 0.4374 | 0.0788 |
| 440 | newly | 0.0099 | 0.1462 | 0.8439 | 869 | newly | 0.4838 | 0.4374 | 0.0788 |
| 912 | newly | 0.0099 | 0.1462 | 0.8439 | 67 | newly | 0.0079 | 0.978 | 0.0141 |
| 653 | newly | 0.0069 | 0.9931 | 0 | 201 | newly | 0.0079 | 0.978 | 0.0141 |
| 451 | newly | 0.0069 | 0.9931 | 0 | 219 | newly | 0.0079 | 0.978 | 0.0141 |
| 158 | newly | 0.6708 | 0.0063 | 0.3229 | 378 | newly | 0.0079 | 0.978 | 0.0141 |
| 26 | newly | 0.6708 | 0.0063 | 0.3229 | 969 | newly | 0.0079 | 0.978 | 0.0141 |
| 704 | newly | 0.6708 | 0.0063 | 0.3229 | 454 | newly | 0.0079 | 0.978 | 0.0141 |
| 652 | newly | 0.3026 | 0.2652 | 0.4323 | 251 | newly | 0.0079 | 0.978 | 0.0141 |
| 226 | newly | 0.1048 | 0.8952 | 0 | 582 | newly | 0.0008 | 0.9992 | 0 |
| 949 | newly | 0.0074 | 0.8785 | 0.1142 | 863 | newly | 0.0008 | 0.9992 | 0 |
| 392 | newly | 0.0074 | 0.8785 | 0.1142 | 437 | newly | 0.0008 | 0.9992 | 0 |
| 866 | newly | 0.0009 | 0.9991 | 0 | 514 | newly | 0.0008 | 0.9992 | 0 |

**Table S6. Probability of type of PrEP and risk of HIV infection among Asian MSM who were using PrEP in the last six months**

|  | **Class 1**  **Informed risk taker (32.9)** | **Class 2**  **On-demand, safe player (68.1)** |
| --- | --- | --- |
| **Probability of** |  |  |
| PrEP type (0=daily, 1=on-demand) | 0.36 | 0.77 |
| Had more than one sexual partner in the last six months | 0.95 | 0.21 |
| Had condomless anal sex with a casual sex partner in the last six months | 0.73 | 0.03 |
| Had an STI diagnosis in the last twelve months | 0.30 | 0.04 |

Akaike’s information criterion = 2,908.583

Bayesian information criterion = 2,951.500

*STI=sexually transmitted infection, PrEP= Pre-exposure prophylaxis for HIV

**Table S7. Probability of each individual in each class for latent class for type of PrEP and risk of HIV infection**

| **ID** | **newly** | **cpr1** | **cpr2** |  | **ID** | **newly** | **cpr1** | **cpr2** |
| --- | --- | --- | --- | --- | --- | --- | --- | --- |
| 374 | No | 0.017122 | 0.982878 |  | 527 | No | 0.0063 | 0.993701 |
| 159 | No | 0.017122 | 0.982878 |  | 24 | No | 0.0063 | 0.993701 |
| 900 | No | 0.017122 | 0.982878 |  | 694 | No | 0.0063 | 0.993701 |
| 711 | No | 0.017122 | 0.982878 |  | 176 | No | 0.0063 | 0.993701 |
| 744 | No | 0.017122 | 0.982878 |  | 445 | No | 0.0063 | 0.993701 |
| 503 | No | 0.017122 | 0.982878 |  | 411 | No | 0.0063 | 0.993701 |
| 854 | No | 0.017122 | 0.982878 |  | 3 | No | 0.0063 | 0.993701 |
| 370 | No | 0.017122 | 0.982878 |  | 814 | No | 0.0063 | 0.993701 |
| 209 | newly | 0.017122 | 0.982878 |  | 719 | No | 0.0063 | 0.993701 |
| 737 | No | 0.017122 | 0.982878 |  | 879 | No | 0.0063 | 0.993701 |
| 532 | No | 0.017122 | 0.982878 |  | 348 | No | 0.0063 | 0.993701 |
| 128 | No | 0.017122 | 0.982878 |  | 308 | newly | 0.0063 | 0.993701 |
| 816 | No | 0.017122 | 0.982878 |  | 230 | newly | 0.0063 | 0.993701 |
| 113 | No | 0.017122 | 0.982878 |  | 222 | No | 0.0063 | 0.993701 |
| 301 | No | 0.017122 | 0.982878 |  | 469 | No | 0.0063 | 0.993701 |
| 380 | No | 0.017122 | 0.982878 |  | 402 | newly | 0.0063 | 0.993701 |
| 44 | No | 0.017122 | 0.982878 |  | 911 | No | 0.0063 | 0.993701 |
| 248 | No | 0.017122 | 0.982878 |  | 778 | No | 0.0063 | 0.993701 |
| 655 | No | 0.137109 | 0.862892 |  | 925 | newly | 0.0063 | 0.993701 |
| 178 | No | 0.137109 | 0.862892 |  | 643 | No | 0.0063 | 0.993701 |
| 288 | newly | 0.137109 | 0.862892 |  | 768 | No | 0.0063 | 0.993701 |
| 144 | newly | 0.137109 | 0.862892 |  | 157 | No | 0.0063 | 0.993701 |
| 670 | No | 0.137109 | 0.862892 |  | 421 | No | 0.0063 | 0.993701 |
| 278 | No | 0.594463 | 0.405537 |  | 275 | No | 0.0063 | 0.993701 |
| 347 | No | 0.594463 | 0.405537 |  | 759 | No | 0.0063 | 0.993701 |
| 272 | No | 0.594463 | 0.405537 |  | 109 | No | 0.0063 | 0.993701 |
| 915 | No | 0.930413 | 0.069587 |  | 217 | No | 0.0063 | 0.993701 |
| 789 | No | 0.544016 | 0.455985 |  | 483 | No | 0.0063 | 0.993701 |
| 496 | No | 0.544016 | 0.455985 |  | 202 | newly | 0.0063 | 0.993701 |
| 748 | No | 0.544016 | 0.455985 |  | 130 | No | 0.0063 | 0.993701 |
| 524 | No | 0.544016 | 0.455985 |  | 813 | No | 0.0063 | 0.993701 |
| 629 | No | 0.544016 | 0.455985 |  | 337 | No | 0.0063 | 0.993701 |
| 302 | No | 0.544016 | 0.455985 |  | 315 | No | 0.0063 | 0.993701 |
| 291 | No | 0.544016 | 0.455985 |  | 18 | No | 0.0063 | 0.993701 |
| 927 | No | 0.544016 | 0.455985 |  | 382 | No | 0.0063 | 0.993701 |
| 892 | No | 0.544016 | 0.455985 |  | 192 | No | 0.0063 | 0.993701 |
| 389 | No | 0.544016 | 0.455985 |  | 6 | newly | 0.0063 | 0.993701 |
| 422 | newly | 0.544016 | 0.455985 |  | 449 | No | 0.0063 | 0.993701 |
| 869 | newly | 0.544016 | 0.455985 |  | 823 | No | 0.0063 | 0.993701 |
| 594 | No | 0.544016 | 0.455985 |  | 965 | newly | 0.0063 | 0.993701 |
| 156 | newly | 0.544016 | 0.455985 |  | 895 | newly | 0.0063 | 0.993701 |
| 349 | No | 0.544016 | 0.455985 |  | 183 | No | 0.0063 | 0.993701 |
| 290 | No | 0.544016 | 0.455985 |  | 959 | No | 0.0063 | 0.993701 |
| 515 | newly | 0.544016 | 0.455985 |  | 78 | No | 0.0063 | 0.993701 |
| 534 | No | 0.544016 | 0.455985 |  | 152 | No | 0.0063 | 0.993701 |
| 824 | No | 0.544016 | 0.455985 |  | 580 | No | 0.0063 | 0.993701 |
| 652 | newly | 0.544016 | 0.455985 |  | 465 | newly | 0.0063 | 0.993701 |
| 686 | No | 0.544016 | 0.455985 |  | 757 | newly | 0.0063 | 0.993701 |
| 521 | No | 0.544016 | 0.455985 |  | 269 | No | 0.0063 | 0.993701 |
| 110 | newly | 0.91584 | 0.08416 |  | 751 | No | 0.0063 | 0.993701 |
| 15 | No | 0.91584 | 0.08416 |  | 433 | No | 0.0063 | 0.993701 |
| 377 | No | 0.91584 | 0.08416 |  | 108 | newly | 0.0063 | 0.993701 |
| 65 | newly | 0.91584 | 0.08416 |  | 430 | No | 0.0063 | 0.993701 |
| 226 | newly | 0.91584 | 0.08416 |  | 628 | newly | 0.0063 | 0.993701 |
| 798 | No | 0.91584 | 0.08416 |  | 583 | newly | 0.0063 | 0.993701 |
| 480 | No | 0.91584 | 0.08416 |  | 136 | newly | 0.0063 | 0.993701 |
| 839 | newly | 0.91584 | 0.08416 |  | 940 | No | 0.0063 | 0.993701 |
| 533 | No | 0.990137 | 0.009863 |  | 236 | No | 0.0063 | 0.993701 |
| 390 | newly | 0.990137 | 0.009863 |  | 254 | No | 0.0063 | 0.993701 |
| 251 | newly | 0.990137 | 0.009863 |  | 701 | newly | 0.0063 | 0.993701 |
| 378 | newly | 0.990137 | 0.009863 |  | 725 | No | 0.0063 | 0.993701 |
| 354 | No | 0.990137 | 0.009863 |  | 623 | No | 0.0063 | 0.993701 |
| 567 | No | 0.990137 | 0.009863 |  | 53 | No | 0.0063 | 0.993701 |
| 381 | No | 0.990137 | 0.009863 |  | 360 | No | 0.0063 | 0.993701 |
| 95 | No | 0.990137 | 0.009863 |  | 151 | No | 0.0063 | 0.993701 |
| 101 | No | 0.990137 | 0.009863 |  | 616 | No | 0.0063 | 0.993701 |
| 542 | newly | 0.990137 | 0.009863 |  | 224 | No | 0.0063 | 0.993701 |
| 321 | No | 0.990137 | 0.009863 |  | 562 | newly | 0.0063 | 0.993701 |
| 219 | newly | 0.990137 | 0.009863 |  | 710 | No | 0.0063 | 0.993701 |
| 97 | No | 0.990137 | 0.009863 |  | 549 | No | 0.0063 | 0.993701 |
| 392 | newly | 0.990137 | 0.009863 |  | 687 | No | 0.0063 | 0.993701 |
| 98 | No | 0.990137 | 0.009863 |  | 256 | No | 0.0063 | 0.993701 |
| 535 | No | 0.990137 | 0.009863 |  | 343 | No | 0.0063 | 0.993701 |
| 578 | No | 0.990137 | 0.009863 |  | 504 | newly | 0.0063 | 0.993701 |
| 369 | No | 0.990137 | 0.009863 |  | 124 | No | 0.0063 | 0.993701 |
| 799 | No | 0.990137 | 0.009863 |  | 490 | No | 0.0063 | 0.993701 |
| 199 | No | 0.990137 | 0.009863 |  | 780 | No | 0.0063 | 0.993701 |
| 454 | newly | 0.990137 | 0.009863 |  | 783 | No | 0.0063 | 0.993701 |
| 873 | newly | 0.990137 | 0.009863 |  | 424 | No | 0.0063 | 0.993701 |
| 657 | newly | 0.990137 | 0.009863 |  | 842 | No | 0.0063 | 0.993701 |
| 63 | No | 0.990137 | 0.009863 |  | 636 | No | 0.0063 | 0.993701 |
| 335 | No | 0.990137 | 0.009863 |  | 934 | No | 0.0063 | 0.993701 |
| 703 | No | 0.990137 | 0.009863 |  | 16 | No | 0.0063 | 0.993701 |
| 821 | No | 0.990137 | 0.009863 |  | 29 | No | 0.0063 | 0.993701 |
| 419 | No | 0.990137 | 0.009863 |  | 868 | No | 0.0063 | 0.993701 |
| 949 | newly | 0.990137 | 0.009863 |  | 286 | No | 0.0063 | 0.993701 |
| 416 | No | 0.990137 | 0.009863 |  | 129 | newly | 0.0063 | 0.993701 |
| 456 | No | 0.990137 | 0.009863 |  | 607 | No | 0.0063 | 0.993701 |
| 453 | No | 0.990137 | 0.009863 |  | 436 | No | 0.0063 | 0.993701 |
| 807 | No | 0.990137 | 0.009863 |  | 92 | No | 0.0063 | 0.993701 |
| 969 | newly | 0.990137 | 0.009863 |  | 231 | No | 0.0063 | 0.993701 |
| 716 | newly | 0.990137 | 0.009863 |  | 689 | No | 0.0063 | 0.993701 |
| 943 | No | 0.990137 | 0.009863 |  | 185 | No | 0.0063 | 0.993701 |
| 353 | No | 0.990137 | 0.009863 |  | 364 | newly | 0.0063 | 0.993701 |
| 941 | No | 0.990137 | 0.009863 |  | 42 | newly | 0.0063 | 0.993701 |
| 121 | newly | 0.990137 | 0.009863 |  | 99 | No | 0.0063 | 0.993701 |
| 67 | newly | 0.990137 | 0.009863 |  | 649 | No | 0.0063 | 0.993701 |
| 693 | newly | 0.990137 | 0.009863 |  | 332 | No | 0.0063 | 0.993701 |
| 804 | newly | 0.990137 | 0.009863 |  | 859 | No | 0.0063 | 0.993701 |
| 116 | No | 0.990137 | 0.009863 |  | 460 | No | 0.0063 | 0.993701 |
| 261 | No | 0.990137 | 0.009863 |  | 255 | No | 0.0063 | 0.993701 |
| 403 | No | 0.998909 | 0.001091 |  | 651 | newly | 0.0063 | 0.993701 |
| 40 | No | 0.998909 | 0.001091 |  | 568 | No | 0.0063 | 0.993701 |
| 518 | newly | 0.998909 | 0.001091 |  | 837 | No | 0.0063 | 0.993701 |
| 875 | No | 0.998909 | 0.001091 |  | 589 | No | 0.0063 | 0.993701 |
| 819 | No | 0.998909 | 0.001091 |  | 494 | newly | 0.0063 | 0.993701 |
| 777 | No | 0.998909 | 0.001091 |  | 794 | No | 0.0063 | 0.993701 |
| 561 | No | 0.998909 | 0.001091 |  | 728 | No | 0.0063 | 0.993701 |
| 90 | No | 0.998909 | 0.001091 |  | 118 | No | 0.0063 | 0.993701 |
| 863 | newly | 0.998909 | 0.001091 |  | 238 | No | 0.0063 | 0.993701 |
| 673 | No | 0.998909 | 0.001091 |  | 318 | newly | 0.0063 | 0.993701 |
| 729 | No | 0.998909 | 0.001091 |  | 640 | No | 0.0063 | 0.993701 |
| 507 | No | 0.998909 | 0.001091 |  | 418 | No | 0.0063 | 0.993701 |
| 860 | newly | 0.998909 | 0.001091 |  | 246 | No | 0.0063 | 0.993701 |
| 582 | newly | 0.998909 | 0.001091 |  | 179 | No | 0.0063 | 0.993701 |
| 866 | newly | 0.998909 | 0.001091 |  | 342 | No | 0.0063 | 0.993701 |
| 587 | No | 0.998909 | 0.001091 |  | 633 | No | 0.0063 | 0.993701 |
| 169 | No | 0.998909 | 0.001091 |  | 536 | No | 0.0063 | 0.993701 |
| 886 | No | 0.998909 | 0.001091 |  | 28 | No | 0.0063 | 0.993701 |
| 890 | No | 0.998909 | 0.001091 |  | 724 | No | 0.0063 | 0.993701 |
| 717 | No | 0.998909 | 0.001091 |  | 512 | No | 0.0063 | 0.993701 |
| 385 | No | 0.998909 | 0.001091 |  | 491 | No | 0.0063 | 0.993701 |
| 572 | No | 0.998909 | 0.001091 |  | 94 | No | 0.0063 | 0.993701 |
| 663 | newly | 0.998909 | 0.001091 |  | 64 | No | 0.0063 | 0.993701 |
| 328 | No | 0.998909 | 0.001091 |  | 513 | newly | 0.0063 | 0.993701 |
| 514 | newly | 0.998909 | 0.001091 |  | 89 | newly | 0.0063 | 0.993701 |
| 327 | No | 0.998909 | 0.001091 |  | 688 | No | 0.0063 | 0.993701 |
| 56 | No | 0.998909 | 0.001091 |  | 803 | No | 0.0063 | 0.993701 |
| 855 | No | 0.998909 | 0.001091 |  | 739 | No | 0.0063 | 0.993701 |
| 46 | No | 0.998909 | 0.001091 |  | 17 | newly | 0.0063 | 0.993701 |
| 21 | No | 0.998909 | 0.001091 |  | 734 | No | 0.0063 | 0.993701 |
| 705 | No | 0.998909 | 0.001091 |  | 475 | No | 0.0063 | 0.993701 |
| 840 | No | 0.998909 | 0.001091 |  | 708 | newly | 0.0063 | 0.993701 |
| 550 | No | 0.998909 | 0.001091 |  | 584 | No | 0.0063 | 0.993701 |
| 203 | No | 0.998909 | 0.001091 |  | 834 | No | 0.0063 | 0.993701 |
| 787 | No | 0.998909 | 0.001091 |  | 193 | newly | 0.0063 | 0.993701 |
| 437 | newly | 0.998909 | 0.001091 |  | 599 | No | 0.0063 | 0.993701 |
| 371 | newly | 0.998909 | 0.001091 |  | 285 | No | 0.0063 | 0.993701 |
| 106 | newly | 0.00297 | 0.997031 |  | 146 | newly | 0.0063 | 0.993701 |
| 704 | newly | 0.00297 | 0.997031 |  | 120 | No | 0.0063 | 0.993701 |
| 957 | newly | 0.00297 | 0.997031 |  | 526 | No | 0.0063 | 0.993701 |
| 270 | No | 0.00297 | 0.997031 |  | 376 | No | 0.0063 | 0.993701 |
| 303 | No | 0.00297 | 0.997031 |  | 147 | No | 0.0063 | 0.993701 |
| 908 | newly | 0.00297 | 0.997031 |  | 611 | No | 0.0063 | 0.993701 |
| 812 | No | 0.00297 | 0.997031 |  | 30 | No | 0.0063 | 0.993701 |
| 186 | No | 0.00297 | 0.997031 |  | 756 | No | 0.0063 | 0.993701 |
| 818 | No | 0.00297 | 0.997031 |  | 443 | No | 0.0063 | 0.993701 |
| 484 | No | 0.00297 | 0.997031 |  | 822 | No | 0.0063 | 0.993701 |
| 237 | No | 0.00297 | 0.997031 |  | 75 | No | 0.0063 | 0.993701 |
| 266 | No | 0.00297 | 0.997031 |  | 921 | No | 0.0063 | 0.993701 |
| 312 | No | 0.00297 | 0.997031 |  | 674 | No | 0.0063 | 0.993701 |
| 617 | No | 0.00297 | 0.997031 |  | 85 | newly | 0.0063 | 0.993701 |
| 481 | No | 0.00297 | 0.997031 |  | 276 | No | 0.0063 | 0.993701 |
| 167 | No | 0.00297 | 0.997031 |  | 408 | No | 0.0063 | 0.993701 |
| 618 | No | 0.00297 | 0.997031 |  | 615 | No | 0.0063 | 0.993701 |
| 135 | No | 0.00297 | 0.997031 |  | 659 | newly | 0.0063 | 0.993701 |
| 463 | No | 0.00297 | 0.997031 |  | 431 | newly | 0.0063 | 0.993701 |
| 281 | No | 0.00297 | 0.997031 |  | 718 | newly | 0.0063 | 0.993701 |
| 277 | No | 0.00297 | 0.997031 |  | 581 | newly | 0.0063 | 0.993701 |
| 131 | No | 0.00297 | 0.997031 |  | 27 | No | 0.0063 | 0.993701 |
| 909 | No | 0.00297 | 0.997031 |  | 409 | newly | 0.0063 | 0.993701 |
| 115 | No | 0.00297 | 0.997031 |  | 229 | No | 0.0063 | 0.993701 |
| 880 | No | 0.00297 | 0.997031 |  | 970 | No | 0.0063 | 0.993701 |
| 225 | No | 0.00297 | 0.997031 |  | 141 | No | 0.0063 | 0.993701 |
| 459 | newly | 0.00297 | 0.997031 |  | 522 | No | 0.0063 | 0.993701 |
| 529 | No | 0.00297 | 0.997031 |  | 373 | newly | 0.0063 | 0.993701 |
| 952 | newly | 0.00297 | 0.997031 |  | 486 | No | 0.0063 | 0.993701 |
| 4 | No | 0.00297 | 0.997031 |  | 37 | No | 0.0063 | 0.993701 |
| 712 | No | 0.00297 | 0.997031 |  | 645 | No | 0.0063 | 0.993701 |
| 316 | No | 0.00297 | 0.997031 |  | 196 | No | 0.0063 | 0.993701 |
| 197 | No | 0.00297 | 0.997031 |  | 334 | No | 0.0063 | 0.993701 |
| 601 | No | 0.00297 | 0.997031 |  | 77 | No | 0.0063 | 0.993701 |
| 761 | No | 0.00297 | 0.997031 |  | 87 | No | 0.0063 | 0.993701 |
| 662 | No | 0.00297 | 0.997031 |  | 425 | newly | 0.0063 | 0.993701 |
| 576 | No | 0.00297 | 0.997031 |  | 896 | newly | 0.0063 | 0.993701 |
| 559 | No | 0.00297 | 0.997031 |  | 307 | No | 0.0063 | 0.993701 |
| 35 | No | 0.00297 | 0.997031 |  | 14 | No | 0.0063 | 0.993701 |
| 498 | No | 0.00297 | 0.997031 |  | 961 | No | 0.0063 | 0.993701 |
| 100 | No | 0.00297 | 0.997031 |  | 937 | No | 0.0063 | 0.993701 |
| 206 | No | 0.00297 | 0.997031 |  | 362 | newly | 0.0063 | 0.993701 |
| 752 | No | 0.00297 | 0.997031 |  | 473 | No | 0.0063 | 0.993701 |
| 545 | No | 0.00297 | 0.997031 |  | 298 | No | 0.0063 | 0.993701 |
| 69 | No | 0.00297 | 0.997031 |  | 309 | No | 0.0063 | 0.993701 |
| 220 | No | 0.00297 | 0.997031 |  | 434 | No | 0.0063 | 0.993701 |
| 606 | No | 0.00297 | 0.997031 |  | 671 | No | 0.0063 | 0.993701 |
| 457 | No | 0.00297 | 0.997031 |  | 153 | No | 0.0063 | 0.993701 |
| 330 | No | 0.00297 | 0.997031 |  | 282 | newly | 0.0063 | 0.993701 |
| 158 | newly | 0.00297 | 0.997031 |  | 356 | No | 0.0063 | 0.993701 |
| 964 | No | 0.00297 | 0.997031 |  | 625 | No | 0.0063 | 0.993701 |
| 852 | No | 0.00297 | 0.997031 |  | 962 | No | 0.0063 | 0.993701 |
| 902 | No | 0.00297 | 0.997031 |  | 573 | newly | 0.0063 | 0.993701 |
| 164 | No | 0.00297 | 0.997031 |  | 923 | newly | 0.0063 | 0.993701 |
| 31 | No | 0.00297 | 0.997031 |  | 665 | No | 0.0063 | 0.993701 |
| 476 | No | 0.00297 | 0.997031 |  | 399 | No | 0.0063 | 0.993701 |
| 472 | No | 0.00297 | 0.997031 |  | 792 | No | 0.0063 | 0.993701 |
| 470 | No | 0.00297 | 0.997031 |  | 888 | newly | 0.0063 | 0.993701 |
| 160 | No | 0.00297 | 0.997031 |  | 557 | No | 0.0063 | 0.993701 |
| 279 | No | 0.00297 | 0.997031 |  | 547 | newly | 0.0063 | 0.993701 |
| 13 | newly | 0.00297 | 0.997031 |  | 259 | No | 0.0063 | 0.993701 |
| 546 | No | 0.00297 | 0.997031 |  | 523 | newly | 0.0063 | 0.993701 |
| 62 | No | 0.00297 | 0.997031 |  | 114 | No | 0.0063 | 0.993701 |
| 551 | No | 0.00297 | 0.997031 |  | 853 | No | 0.0063 | 0.993701 |
| 210 | No | 0.00297 | 0.997031 |  | 619 | No | 0.0063 | 0.993701 |
| 723 | No | 0.00297 | 0.997031 |  | 575 | No | 0.0063 | 0.993701 |
| 932 | No | 0.00297 | 0.997031 |  | 502 | No | 0.0063 | 0.993701 |
| 505 | No | 0.00297 | 0.997031 |  | 404 | newly | 0.0063 | 0.993701 |
| 150 | No | 0.00297 | 0.997031 |  | 917 | No | 0.0063 | 0.993701 |
| 749 | No | 0.00297 | 0.997031 |  | 444 | No | 0.0063 | 0.993701 |
| 953 | No | 0.00297 | 0.997031 |  | 162 | No | 0.0063 | 0.993701 |
| 211 | No | 0.00297 | 0.997031 |  | 936 | No | 0.054663 | 0.945337 |
| 829 | No | 0.00297 | 0.997031 |  | 709 | No | 0.054663 | 0.945337 |
| 806 | No | 0.00297 | 0.997031 |  | 477 | No | 0.054663 | 0.945337 |
| 910 | No | 0.00297 | 0.997031 |  | 340 | No | 0.054663 | 0.945337 |
| 592 | No | 0.00297 | 0.997031 |  | 609 | No | 0.054663 | 0.945337 |
| 66 | No | 0.00297 | 0.997031 |  | 509 | No | 0.054663 | 0.945337 |
| 918 | No | 0.00297 | 0.997031 |  | 133 | newly | 0.054663 | 0.945337 |
| 544 | No | 0.00297 | 0.997031 |  | 12 | No | 0.054663 | 0.945337 |
| 685 | No | 0.00297 | 0.997031 |  | 263 | No | 0.054663 | 0.945337 |
| 26 | newly | 0.00297 | 0.997031 |  | 926 | No | 0.054663 | 0.945337 |
| 143 | No | 0.00297 | 0.997031 |  | 699 | newly | 0.054663 | 0.945337 |
| 329 | No | 0.00297 | 0.997031 |  | 781 | No | 0.054663 | 0.945337 |
| 691 | No | 0.026448 | 0.973552 |  | 81 | No | 0.054663 | 0.945337 |
| 274 | No | 0.026448 | 0.973552 |  | 122 | No | 0.054663 | 0.945337 |
| 182 | No | 0.026448 | 0.973552 |  | 857 | No | 0.347874 | 0.652126 |
| 864 | newly | 0.026448 | 0.973552 |  | 177 | newly | 0.347874 | 0.652126 |
| 2 | No | 0.200397 | 0.799604 |  | 495 | No | 0.347874 | 0.652126 |
| 845 | newly | 0.200397 | 0.799604 |  | 646 | No | 0.347874 | 0.652126 |
| 351 | No | 0.200397 | 0.799604 |  | 553 | newly | 0.347874 | 0.652126 |
| 119 | newly | 0.695675 | 0.304325 |  | 166 | No | 0.347874 | 0.652126 |
| 417 | No | 0.16942 | 0.83058 |  | 76 | newly | 0.347874 | 0.652126 |
| 289 | No | 0.16942 | 0.83058 |  | 139 | No | 0.347874 | 0.652126 |
| 774 | newly | 0.16942 | 0.83058 |  | 844 | No | 0.347874 | 0.652126 |
| 935 | No | 0.16942 | 0.83058 |  | 669 | No | 0.347874 | 0.652126 |
| 123 | No | 0.16942 | 0.83058 |  | 79 | No | 0.347874 | 0.652126 |
| 420 | No | 0.16942 | 0.83058 |  | 585 | newly | 0.347874 | 0.652126 |
| 20 | No | 0.16942 | 0.83058 |  | 945 | newly | 0.347874 | 0.652126 |
| 817 | No | 0.16942 | 0.83058 |  | 608 | No | 0.347874 | 0.652126 |
| 922 | No | 0.16942 | 0.83058 |  | 702 | No | 0.347874 | 0.652126 |
| 52 | No | 0.16942 | 0.83058 |  | 117 | No | 0.347874 | 0.652126 |
| 11 | No | 0.16942 | 0.83058 |  | 468 | No | 0.829517 | 0.170483 |
| 849 | No | 0.16942 | 0.83058 |  | 732 | No | 0.302732 | 0.697268 |
| 944 | No | 0.16942 | 0.83058 |  | 319 | newly | 0.302732 | 0.697268 |
| 233 | No | 0.16942 | 0.83058 |  | 627 | newly | 0.302732 | 0.697268 |
| 300 | No | 0.16942 | 0.83058 |  | 764 | newly | 0.302732 | 0.697268 |
| 931 | No | 0.16942 | 0.83058 |  | 432 | No | 0.302732 | 0.697268 |
| 359 | newly | 0.16942 | 0.83058 |  | 510 | newly | 0.302732 | 0.697268 |
| 884 | No | 0.16942 | 0.83058 |  | 713 | No | 0.302732 | 0.697268 |
| 398 | No | 0.16942 | 0.83058 |  | 244 | No | 0.302732 | 0.697268 |
| 809 | No | 0.16942 | 0.83058 |  | 384 | newly | 0.302732 | 0.697268 |
| 598 | No | 0.16942 | 0.83058 |  | 668 | No | 0.302732 | 0.697268 |
| 391 | No | 0.16942 | 0.83058 |  | 25 | newly | 0.302732 | 0.697268 |
| 916 | No | 0.16942 | 0.83058 |  | 613 | newly | 0.302732 | 0.697268 |
| 258 | No | 0.16942 | 0.83058 |  | 569 | No | 0.302732 | 0.697268 |
| 637 | No | 0.16942 | 0.83058 |  | 574 | No | 0.302732 | 0.697268 |
| 388 | No | 0.650414 | 0.349586 |  | 426 | No | 0.302732 | 0.697268 |
| 287 | No | 0.650414 | 0.349586 |  | 753 | No | 0.302732 | 0.697268 |
| 742 | newly | 0.650414 | 0.349586 |  | 745 | No | 0.302732 | 0.697268 |
| 104 | No | 0.650414 | 0.349586 |  | 681 | No | 0.302732 | 0.697268 |
| 407 | No | 0.944946 | 0.055054 |  | 610 | No | 0.302732 | 0.697268 |
| 791 | No | 0.944946 | 0.055054 |  | 602 | newly | 0.302732 | 0.697268 |
| 138 | No | 0.944946 | 0.055054 |  | 245 | No | 0.302732 | 0.697268 |
| 786 | newly | 0.944946 | 0.055054 |  | 590 | No | 0.302732 | 0.697268 |
| 161 | No | 0.944946 | 0.055054 |  | 556 | No | 0.302732 | 0.697268 |
| 72 | No | 0.944946 | 0.055054 |  | 366 | No | 0.302732 | 0.697268 |
| 355 | newly | 0.944946 | 0.055054 |  | 19 | No | 0.302732 | 0.697268 |
| 773 | No | 0.944946 | 0.055054 |  | 50 | No | 0.302732 | 0.697268 |
| 395 | No | 0.944946 | 0.055054 |  | 127 | newly | 0.302732 | 0.697268 |
| 788 | No | 0.944946 | 0.055054 |  | 808 | newly | 0.302732 | 0.697268 |
| 485 | No | 0.944946 | 0.055054 |  | 396 | newly | 0.302732 | 0.697268 |
| 730 | No | 0.944946 | 0.055054 |  | 397 | No | 0.302732 | 0.697268 |
| 815 | No | 0.944946 | 0.055054 |  | 295 | No | 0.302732 | 0.697268 |
| 482 | No | 0.944946 | 0.055054 |  | 221 | No | 0.302732 | 0.697268 |
| 800 | No | 0.944946 | 0.055054 |  | 914 | No | 0.302732 | 0.697268 |
| 201 | newly | 0.944946 | 0.055054 |  | 350 | No | 0.302732 | 0.697268 |
| 667 | newly | 0.944946 | 0.055054 |  | 555 | No | 0.302732 | 0.697268 |
| 648 | No | 0.944946 | 0.055054 |  | 441 | No | 0.302732 | 0.697268 |
| 82 | No | 0.944946 | 0.055054 |  | 458 | newly | 0.302732 | 0.697268 |
| 142 | No | 0.944946 | 0.055054 |  | 672 | newly | 0.302732 | 0.697268 |
| 680 | No | 0.944946 | 0.055054 |  | 654 | No | 0.302732 | 0.697268 |
| 692 | No | 0.944946 | 0.055054 |  | 947 | No | 0.302732 | 0.697268 |
| 194 | No | 0.944946 | 0.055054 |  | 214 | No | 0.302732 | 0.697268 |
| 311 | No | 0.944946 | 0.055054 |  | 554 | No | 0.302732 | 0.697268 |
| 207 | No | 0.944946 | 0.055054 |  | 540 | newly | 0.302732 | 0.697268 |
| 811 | No | 0.944946 | 0.055054 |  | 428 | newly | 0.302732 | 0.697268 |
| 415 | No | 0.944946 | 0.055054 |  | 738 | No | 0.302732 | 0.697268 |
| 464 | No | 0.944946 | 0.055054 |  | 163 | No | 0.302732 | 0.697268 |
| 963 | No | 0.944946 | 0.055054 |  | 832 | No | 0.302732 | 0.697268 |
| 511 | No | 0.944946 | 0.055054 |  | 48 | No | 0.302732 | 0.697268 |
| 216 | No | 0.944946 | 0.055054 |  | 907 | No | 0.302732 | 0.697268 |
| 904 | No | 0.944946 | 0.055054 |  | 184 | newly | 0.302732 | 0.697268 |
| 825 | No | 0.944946 | 0.055054 |  | 429 | No | 0.302732 | 0.697268 |
| 487 | No | 0.944946 | 0.055054 |  | 212 | No | 0.302732 | 0.697268 |
| 620 | No | 0.944946 | 0.055054 |  | 851 | No | 0.302732 | 0.697268 |
| 341 | newly | 0.944946 | 0.055054 |  | 638 | newly | 0.302732 | 0.697268 |
| 386 | No | 0.944946 | 0.055054 |  | 467 | No | 0.302732 | 0.697268 |
| 379 | No | 0.993653 | 0.006347 |  | 740 | No | 0.302732 | 0.697268 |
| 950 | No | 0.993653 | 0.006347 |  | 955 | No | 0.302732 | 0.697268 |
| 658 | No | 0.993653 | 0.006347 |  | 564 | newly | 0.302732 | 0.697268 |
| 107 | No | 0.993653 | 0.006347 |  | 59 | No | 0.302732 | 0.697268 |
| 530 | No | 0.993653 | 0.006347 |  | 960 | No | 0.302732 | 0.697268 |
| 501 | No | 0.993653 | 0.006347 |  | 383 | No | 0.302732 | 0.697268 |
| 604 | No | 0.993653 | 0.006347 |  | 913 | No | 0.302732 | 0.697268 |
| 450 | newly | 0.993653 | 0.006347 |  | 600 | No | 0.302732 | 0.697268 |
| 750 | newly | 0.993653 | 0.006347 |  | 707 | No | 0.302732 | 0.697268 |
| 280 | No | 0.993653 | 0.006347 |  | 765 | No | 0.302732 | 0.697268 |
| 565 | newly | 0.993653 | 0.006347 |  | 223 | No | 0.302732 | 0.697268 |
| 596 | No | 0.993653 | 0.006347 |  | 305 | No | 0.302732 | 0.697268 |
| 145 | No | 0.993653 | 0.006347 |  | 684 | No | 0.302732 | 0.697268 |
| 427 | No | 0.993653 | 0.006347 |  | 621 | No | 0.302732 | 0.697268 |
| 297 | No | 0.993653 | 0.006347 |  | 721 | No | 0.302732 | 0.697268 |
| 967 | No | 0.0063 | 0.993701 |  | 54 | No | 0.302732 | 0.697268 |
| 126 | No | 0.0063 | 0.993701 |  | 858 | No | 0.302732 | 0.697268 |
| 363 | newly | 0.0063 | 0.993701 |  | 696 | No | 0.302732 | 0.697268 |
| 543 | No | 0.0063 | 0.993701 |  | 466 | No | 0.302732 | 0.697268 |
| 293 | No | 0.0063 | 0.993701 |  | 447 | No | 0.302732 | 0.697268 |
| 412 | No | 0.0063 | 0.993701 |  | 41 | newly | 0.302732 | 0.697268 |
| 614 | No | 0.0063 | 0.993701 |  | 111 | No | 0.302732 | 0.697268 |
| 125 | newly | 0.0063 | 0.993701 |  | 172 | No | 0.302732 | 0.697268 |
| 51 | No | 0.0063 | 0.993701 |  | 843 | No | 0.302732 | 0.697268 |
| 642 | No | 0.0063 | 0.993701 |  | 304 | No | 0.302732 | 0.697268 |
| 338 | newly | 0.0063 | 0.993701 |  | 836 | newly | 0.302732 | 0.697268 |
| 45 | No | 0.0063 | 0.993701 |  | 265 | No | 0.302732 | 0.697268 |
| 664 | No | 0.0063 | 0.993701 |  | 570 | No | 0.302732 | 0.697268 |
| 96 | newly | 0.0063 | 0.993701 |  | 492 | newly | 0.302732 | 0.697268 |
| 508 | No | 0.0063 | 0.993701 |  | 579 | newly | 0.302732 | 0.697268 |
| 271 | No | 0.0063 | 0.993701 |  | 55 | No | 0.302732 | 0.697268 |
| 867 | No | 0.0063 | 0.993701 |  | 93 | No | 0.302732 | 0.697268 |
| 91 | No | 0.0063 | 0.993701 |  | 455 | No | 0.302732 | 0.697268 |
| 720 | No | 0.0063 | 0.993701 |  | 372 | No | 0.302732 | 0.697268 |
| 571 | newly | 0.0063 | 0.993701 |  | 243 | No | 0.302732 | 0.697268 |
| 394 | No | 0.0063 | 0.993701 |  | 919 | No | 0.302732 | 0.697268 |
| 770 | No | 0.0063 | 0.993701 |  | 622 | No | 0.302732 | 0.697268 |
| 189 | newly | 0.0063 | 0.993701 |  | 435 | No | 0.302732 | 0.697268 |
| 320 | No | 0.0063 | 0.993701 |  | 413 | No | 0.302732 | 0.697268 |
| 49 | No | 0.0063 | 0.993701 |  | 324 | No | 0.302732 | 0.697268 |
| 635 | newly | 0.0063 | 0.993701 |  | 850 | No | 0.302732 | 0.697268 |
| 782 | No | 0.0063 | 0.993701 |  | 299 | No | 0.302732 | 0.697268 |
| 958 | No | 0.0063 | 0.993701 |  | 155 | No | 0.302732 | 0.697268 |
| 213 | No | 0.0063 | 0.993701 |  | 767 | No | 0.302732 | 0.697268 |
| 240 | No | 0.0063 | 0.993701 |  | 939 | No | 0.302732 | 0.697268 |
| 870 | No | 0.0063 | 0.993701 |  | 528 | No | 0.302732 | 0.697268 |
| 891 | No | 0.0063 | 0.993701 |  | 841 | No | 0.302732 | 0.697268 |
| 242 | No | 0.0063 | 0.993701 |  | 187 | newly | 0.302732 | 0.697268 |
| 401 | No | 0.0063 | 0.993701 |  | 331 | No | 0.302732 | 0.697268 |
| 32 | No | 0.0063 | 0.993701 |  | 247 | No | 0.302732 | 0.697268 |
| 683 | No | 0.0063 | 0.993701 |  | 847 | No | 0.302732 | 0.697268 |
| 735 | No | 0.0063 | 0.993701 |  | 848 | No | 0.302732 | 0.697268 |
| 929 | No | 0.0063 | 0.993701 |  | 666 | No | 0.302732 | 0.697268 |
| 36 | newly | 0.0063 | 0.993701 |  | 548 | No | 0.302732 | 0.697268 |
| 313 | newly | 0.0063 | 0.993701 |  | 831 | No | 0.302732 | 0.697268 |
| 80 | No | 0.0063 | 0.993701 |  | 951 | No | 0.302732 | 0.697268 |
| 833 | newly | 0.0063 | 0.993701 |  | 173 | newly | 0.302732 | 0.697268 |
| 326 | No | 0.0063 | 0.993701 |  | 405 | newly | 0.302732 | 0.697268 |
| 22 | No | 0.0063 | 0.993701 |  | 899 | No | 0.302732 | 0.697268 |
| 190 | No | 0.0063 | 0.993701 |  | 743 | No | 0.302732 | 0.697268 |
| 525 | newly | 0.0063 | 0.993701 |  | 84 | No | 0.302732 | 0.697268 |
| 566 | No | 0.0063 | 0.993701 |  | 103 | No | 0.302732 | 0.697268 |
| 901 | No | 0.0063 | 0.993701 |  | 323 | No | 0.302732 | 0.697268 |
| 827 | No | 0.0063 | 0.993701 |  | 112 | No | 0.302732 | 0.697268 |
| 903 | No | 0.0063 | 0.993701 |  | 1 | No | 0.302732 | 0.697268 |
| 367 | No | 0.0063 | 0.993701 |  | 264 | No | 0.302732 | 0.697268 |
| 448 | No | 0.0063 | 0.993701 |  | 560 | No | 0.798393 | 0.201607 |
| 810 | No | 0.0063 | 0.993701 |  | 793 | newly | 0.798393 | 0.201607 |
| 181 | newly | 0.0063 | 0.993701 |  | 758 | No | 0.798393 | 0.201607 |
| 149 | No | 0.0063 | 0.993701 |  | 58 | No | 0.798393 | 0.201607 |
| 200 | newly | 0.0063 | 0.993701 |  | 102 | No | 0.798393 | 0.201607 |
| 260 | No | 0.0063 | 0.993701 |  | 715 | No | 0.798393 | 0.201607 |
| 862 | No | 0.0063 | 0.993701 |  | 234 | No | 0.798393 | 0.201607 |
| 267 | No | 0.0063 | 0.993701 |  | 8 | No | 0.798393 | 0.201607 |
| 478 | No | 0.0063 | 0.993701 |  | 874 | newly | 0.798393 | 0.201607 |
| 563 | No | 0.0063 | 0.993701 |  | 7 | No | 0.973357 | 0.026643 |
| 137 | No | 0.0063 | 0.993701 |  | 39 | No | 0.973357 | 0.026643 |
| 538 | No | 0.0063 | 0.993701 |  | 310 | No | 0.973357 | 0.026643 |
| 954 | No | 0.0063 | 0.993701 |  | 586 | newly | 0.973357 | 0.026643 |
| 375 | newly | 0.0063 | 0.993701 |  | 677 | No | 0.973357 | 0.026643 |
| 802 | No | 0.0063 | 0.993701 |  | 208 | No | 0.973357 | 0.026643 |
| 519 | No | 0.0063 | 0.993701 |  | 314 | newly | 0.973357 | 0.026643 |
| 695 | No | 0.0063 | 0.993701 |  | 775 | No | 0.973357 | 0.026643 |
| 180 | No | 0.0063 | 0.993701 |  | 283 | newly | 0.973357 | 0.026643 |
| 736 | No | 0.0063 | 0.993701 |  | 595 | No | 0.973357 | 0.026643 |
| 872 | No | 0.0063 | 0.993701 |  | 588 | No | 0.973357 | 0.026643 |
| 154 | No | 0.0063 | 0.993701 |  | 506 | No | 0.973357 | 0.026643 |
| 727 | No | 0.0063 | 0.993701 |  | 60 | newly | 0.973357 | 0.026643 |
| 898 | newly | 0.0063 | 0.993701 |  | 755 | No | 0.973357 | 0.026643 |
| 746 | newly | 0.0063 | 0.993701 |  | 660 | No | 0.973357 | 0.026643 |
| 885 | No | 0.0063 | 0.993701 |  | 912 | newly | 0.973357 | 0.026643 |
| 73 | No | 0.0063 | 0.993701 |  | 876 | No | 0.973357 | 0.026643 |
| 471 | newly | 0.0063 | 0.993701 |  | 452 | No | 0.973357 | 0.026643 |
| 593 | No | 0.0063 | 0.993701 |  | 520 | No | 0.973357 | 0.026643 |
| 9 | No | 0.0063 | 0.993701 |  | 499 | No | 0.973357 | 0.026643 |
| 38 | No | 0.0063 | 0.993701 |  | 218 | newly | 0.973357 | 0.026643 |
| 883 | No | 0.0063 | 0.993701 |  | 784 | No | 0.973357 | 0.026643 |
| 772 | No | 0.0063 | 0.993701 |  | 865 | newly | 0.973357 | 0.026643 |
| 83 | newly | 0.0063 | 0.993701 |  | 336 | No | 0.973357 | 0.026643 |
| 797 | No | 0.0063 | 0.993701 |  | 252 | newly | 0.973357 | 0.026643 |
| 345 | No | 0.0063 | 0.993701 |  | 474 | No | 0.973357 | 0.026643 |
| 603 | newly | 0.0063 | 0.993701 |  | 440 | newly | 0.973357 | 0.026643 |
| 552 | No | 0.0063 | 0.993701 |  | 747 | No | 0.973357 | 0.026643 |
| 700 | No | 0.0063 | 0.993701 |  | 497 | No | 0.973357 | 0.026643 |
| 893 | No | 0.0063 | 0.993701 |  | 344 | newly | 0.973357 | 0.026643 |
| 650 | No | 0.0063 | 0.993701 |  | 250 | newly | 0.973357 | 0.026643 |
| 105 | No | 0.0063 | 0.993701 |  | 227 | No | 0.973357 | 0.026643 |
| 292 | No | 0.0063 | 0.993701 |  | 273 | No | 0.973357 | 0.026643 |
| 682 | No | 0.0063 | 0.993701 |  | 828 | No | 0.973357 | 0.026643 |
| 731 | No | 0.0063 | 0.993701 |  | 294 | newly | 0.973357 | 0.026643 |
| 906 | No | 0.0063 | 0.993701 |  | 306 | No | 0.973357 | 0.026643 |
| 956 | No | 0.0063 | 0.993701 |  | 10 | newly | 0.973357 | 0.026643 |
| 195 | No | 0.0063 | 0.993701 |  | 365 | No | 0.973357 | 0.026643 |
| 878 | No | 0.0063 | 0.993701 |  | 541 | No | 0.973357 | 0.026643 |
| 191 | No | 0.0063 | 0.993701 |  | 134 | newly | 0.973357 | 0.026643 |
| 71 | No | 0.0063 | 0.993701 |  | 215 | No | 0.973357 | 0.026643 |
| 439 | No | 0.0063 | 0.993701 |  | 205 | newly | 0.973357 | 0.026643 |
| 795 | No | 0.0063 | 0.993701 |  | 948 | newly | 0.973357 | 0.026643 |
| 726 | newly | 0.0063 | 0.993701 |  | 47 | No | 0.973357 | 0.026643 |
| 771 | newly | 0.0063 | 0.993701 |  | 23 | No | 0.973357 | 0.026643 |
| 897 | No | 0.0063 | 0.993701 |  | 656 | No | 0.973357 | 0.026643 |
| 846 | No | 0.0063 | 0.993701 |  | 639 | No | 0.973357 | 0.026643 |
| 577 | newly | 0.0063 | 0.993701 |  | 741 | No | 0.973357 | 0.026643 |
| 500 | No | 0.0063 | 0.993701 |  | 414 | No | 0.973357 | 0.026643 |
| 204 | No | 0.0063 | 0.993701 |  | 605 | No | 0.973357 | 0.026643 |
| 933 | No | 0.0063 | 0.993701 |  | 400 | No | 0.973357 | 0.026643 |
| 612 | No | 0.0063 | 0.993701 |  | 762 | No | 0.973357 | 0.026643 |
| 61 | No | 0.0063 | 0.993701 |  | 479 | newly | 0.973357 | 0.026643 |
| 317 | newly | 0.0063 | 0.993701 |  | 253 | No | 0.973357 | 0.026643 |
| 235 | No | 0.0063 | 0.993701 |  | 881 | No | 0.973357 | 0.026643 |
| 820 | No | 0.0063 | 0.993701 |  | 631 | No | 0.997008 | 0.002992 |
| 188 | No | 0.0063 | 0.993701 |  | 451 | newly | 0.997008 | 0.002992 |
| 539 | newly | 0.0063 | 0.993701 |  | 262 | No | 0.997008 | 0.002992 |
| 838 | No | 0.0063 | 0.993701 |  | 232 | No | 0.997008 | 0.002992 |
| 68 | No | 0.0063 | 0.993701 |  | 357 | No | 0.997008 | 0.002992 |
| 763 | No | 0.0063 | 0.993701 |  | 630 | No | 0.997008 | 0.002992 |
| 769 | newly | 0.0063 | 0.993701 |  | 826 | No | 0.997008 | 0.002992 |
| 733 | No | 0.0063 | 0.993701 |  | 653 | newly | 0.997008 | 0.002992 |
| 165 | No | 0.0063 | 0.993701 |  | 406 | No | 0.997008 | 0.002992 |
| 174 | No | 0.0063 | 0.993701 |  |  |  |  |  |
| 626 | No | 0.0063 | 0.993701 |  |  |  |  |  |
